# Reducing Coke and Increasing Bio-Oil Yield during Catalytic Fast Pyrolysis of Biomass Using Phosphorus-Modified Zeolite Catalysts

**DOI:** 10.1021/acssuschemeng.5c04731

**Published:** 2025-12-03

**Authors:** Cody J. Wrasman, Brittney E. Petel, Carson Pierce, Kellene A. Orton, Scott Palmer, Jacklyn N. Hall, Hacksung Kim, Fulya Dogan, Theodore R. Krause, Huamin Wang, Xinbin Yu, Stefano Dell’Orco, Kinga A. Unocic, Alexandre C. Foucher, Tomas Grejtak, David A. Cullen, Grace Blaskowski, Frederick G. Baddour, Xiaolin Chen, Kristiina Iisa, Abhijit Dutta, Susan E. Habas, Michael B. Griffin

**Affiliations:** † 53405 National Renewable Energy Laboratory, Golden, Colorado 80401, United States; ‡ 1291 Argonne National Laboratory, Lemont, Illinois 60439, United States; § Northwestern University, Evanston, Illinois 60208, United States; ∥ 6865 Pacific Northwest National Laboratory, Richland, Washington 99352, United States; ⊥ 6146 Oak Ridge National Laboratory, Oak Ridge, Tennessee 37831, United States

**Keywords:** biofuels, biocrude, SAF, ZSM-5, hydrotreating

## Abstract

Catalytic fast pyrolysis (CFP) is a promising strategy for producing hydrocarbon transportation fuels from biomass feedstocks. However, catalyst development is needed to increase bio-oil yields and enhance process economics. In this work, we demonstrate how post synthetic modification of formed ZSM-5 with phosphorus shifts CFP selectivity from coke and light gases toward the desired bio-oil product. Microscale experiments demonstrated reduced coke production relative to unmodified ZSM-5 and identified an optimal P loading. Extensive catalyst characterization revealed that P interacted with Al sites to reduce the acid site density, with preferential binding to the strongest acid sites. Insights from the microscale experiments were leveraged to produce kilogram quantities of formed P-ZSM-5 for evaluation in a larger semi-integrated process. These experiments generated liters of bio-oil that was hydrotreated and fractionated into gasoline, diesel, and jet cuts. The phosphorus-modified ZSM-5 improved CFP bio-oil yield, resulting in an 11% relative increase in the carbon yield from biomass to aviation fuel and a 14% decrease in the minimum fuel selling price. These results highlight the impact targeted changes in catalyst acidity, achieved by adding 2.5 wt % P, can have on the carbon efficiency and feasibility of fuel production from biomass feedstocks.

## Graphical abstract

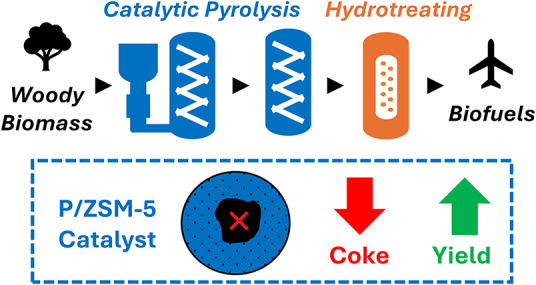

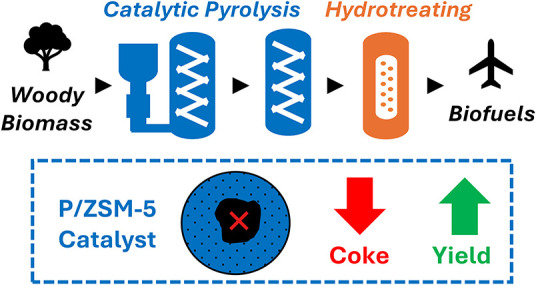

## Introduction

Catalytic fast pyrolysis (CFP) is a promising pathway for the conversion of woody biomass into a stabilized bio-oil that can be hydrotreated into liquid transportation fuels.^1^ This approach serves to increase available energy resources. For example, it is estimated that CFP could produce in excess of 8 billion gallons of hydrocarbon fuels in the U.S. per year based on the projected availability of forest and woody waste resources in the US Department of Energy Billion Ton Report^2^ and previously demonstrated aviation fuel yields.^3^ During CFP, biomass is thermally deconstructed into vapors that are further catalytically deoxygenated to produce bio-oil, an aqueous phase, char, catalyst coke, and permanent gases. The bio-oil can be hydrotreated to remove the remaining oxygen, resulting in a hydrocarbon product that can be fractionated into fuels.^3,4^ Noncatalytic biomass pyrolysis has already been commercialized. However, the resulting highly oxygenated bio-oils are thermally unstable,^5^ and require complex processes, like multistep hydrotreating or other pretreatments, to successfully produce hydrocarbons.^6,7^ The addition of a catalytic step in CFP produces a bio-oil containing less oxygen than fast pyrolysis alone, meaning it can be hydrotreated in a single-step process without intermediate stabilization.^6^

CFP offers high process flexibility and has been investigated in a variety of configurations.^1^ CFP has attracted commercial interest from companies including Anellotech, BioBTX, and KiOR.^1^ Feedstocks as diverse as pine,^3^ crop residues,^8^ and plastics,^9^ have been converted using zeolites,^10^ amorphous oxides,^11^ supported metals,^12^ and transition metal carbide catalysts^13^ in inert or H_2_ atmospheres.^14^ CFP can be conducted in an in situ configuration where the catalyst physically contacts the feedstock,^15^ or in an ex situ configuration where catalytic upgrading occurs in a separate reactor. While the in situ configuration simplifies the process, it risks catalyst deactivation by contaminants like alkali metals in the feedstock. While the ex situ configuration incurs a higher capital cost for multiple reactors, it extends catalyst lifetimes and also allows for optimization of both pyrolysis and catalytic upgrading temperatures. The zeolite ZSM-5 is a common choice for biomass CFP due to its (1) ability to operate without H_2_, (2) coke resistance, and (3) selectivity toward single-ring aromatic products.

Technoeconomic analysis (TEA) indicates that a key factor influencing the fuel selling price in an integrated CFP-hydrotreating process is the bio-oil yield from the CFP step.^16^ CFP bio-oil yields are limited by the unselective nature of thermal feedstock decomposition as well as the abundance of competing nonfuel products produced during catalytic upgrading. Therefore, strategies to modify the CFP catalyst to maximize bio-oil yield while minimizing byproducts like coke are critical.^17^ Zeolite catalysts are good candidates for modification as they upgrade pyrolysis vapors through acid catalyzed cracking reactions,^10^ and they can be fully regenerated by thermal oxidation.^18,19^

Previous studies have shown that adding metals,^14^ increasing mesoporosity,^20^ or adjusting catalyst acidity,^21^ can increase CFP bio-oil yields. Others have also studied doping ZSM-5 catalysts with phosphorus.^22^ Phosphorus-modified ZSM-5 (P-ZSM-5) catalysts have been extensively studied for various applications, including cracking,^23^ methanol-to-olefins,^24,25^ and ethanol dehydration.^26–28^ In these cases, adding phosphorus reduced the strong ZSM-5 acidity through interactions between P and framework Al in acid sites. Decreasing acidity reduced hydrogen transfer reactions, leading to more olefin products,^23–25,27^ and lower catalyst coke formation.^24,25,27^ Phosphorus has also been shown to increase the hydrothermal stability of ZSM-5, preventing dealumination at elevated temperature,^29–31^ and allowing for more catalyst regeneration cycles. Based on this information, we hypothesized that the modification of a typical ZSM-5 CFP catalyst with P could result in improved bio-oil yield and lower coke yield, thus resulting in favorable economic performance.

In this work, we demonstrate the benefits of using P-ZSM-5 in a semi-integrated biomass ex situ CFP-hydrotreating process. We first synthesized a series of P-ZSM-5 catalysts by adding between 1 and 5 wt % P to a formed ZSM-5 catalyst with an Al_2_O_3_ binder. This range of P was selected based on literature results that show coke reduction benefits at P loading up to 3.4 wt %,^26^ and near complete loss of strong acidity, which is needed for biomass CFP, at 6 wt % P.^32^ The catalysts were characterized and evaluated at the microscale to determine hydrocarbon and coke yields. Catalytic evaluation in conjunction with catalyst characterization revealed that P interacts with Al in both the ZSM-5 and the Al_2_O_3_ binder, attenuating the strongest acid sites, leading to a reduced carbon yield to coke and an increased bio-oil productivity. The catalyst with the highest yield of desired products was synthesized at the kg scale and tested in a semi-integrated CFP and hydrotreating process to produce hydrocarbon liquids that were analyzed for their fuel properties. This investigation showed that the addition of P to ZSM-5 led to a decrease in coke production and an increase in bio-oil yield. The larger scale experimental yields were used as the basis for TEA and lifecycle analysis models to assess the influence of catalyst chemistry on CFP process economics and carbon intensity. Overall, the addition of P to ZSM-5 led to a 14% relative decrease in the minimum fuel selling price compared to the unmodified catalyst.

## Methods

### Materials

Formed 0.5 mm ZSM-5 catalyst spheres, provided by Johnson Matthey, were comprised of 80 wt % ZSM-5 with a SiO_2_/Al_2_O_3_ ratio of 30 and 20 wt % Al_2_O_3_ binder. Phosphoric acid (85 wt % in water) was purchased from Sigma-Aldrich. Pine biomass containing 1.5 wt % ash ground to an average particle size of 0.5 mm was provided by Idaho National Laboratory. Ultimate and proximate analysis of the biomass can be found in Table S1, and a particle size distribution can be found in Figure S1.

### Catalyst Synthesis

Formed ZSM-5 catalysts were modified by the addition of 1, 2.5, and 5 wt % P. Modified catalysts are denoted X%-P-ZSM-5 where X represents the nominal mass loading of P. P-ZSM-5 catalysts were synthesized by adding the desired volume of phosphoric acid in water to ZSM-5 particles to reach the incipient wetness point, allowing the material to dry at ambient conditions overnight, followed by drying in a 120 °C furnace overnight. The resulting material was then calcined in flowing air by ramping the temperature at 1 °C/min to 550 °C and holding at that temperature for 3 h.

### Reaction Testing

#### Microreactor Experiments

Catalysts were tested in a Frontier Rx-3050TR tandem microreactor continuously purged with 54 mL/min He. In these experiments, 1 mg biomass was placed at the bottom of a stainless-steel sample cup followed by a quartz filter to separate pyrolyzing biomass from the catalyst. Five mg of catalyst crushed to below 200 μm was then added on top of the filter followed by a second quartz filter to hold all materials in the sample cup. Samples were dropped into the top section of the tandem microreactor held at 500 °C. The resulting upgraded vapors were fed directly into a microjet cryotrap where they were condensed at −196 °C. Permanent gases passed through the cryotrap and entered the gas chromatography (7890B, Agilent Technologies) mass spectrometry (5977A, Agilent Technologies) (GCMS) system equipped with a 5% diphenyl and 95% dimethylpolysiloxane stationary phase capillary column for vapor separation, a flame ionization detector for species quantification, and a thermal conductivity detector for permanent gas identification. Vapors were evaporated from the cryotrap as it was heated with the gas chromatography column at a rate of 20 °C/min from 40 to 300 °C. Products were identified by mass spectrometry using the NIST library and quantified using the flame ionization detector which was calibrated using a mixture of 30 hydrocarbon and oxygenate compounds. This system can quickly sample a variety of catalysts and operating conditions. It is important to note that the GCMS-FID system can only characterize 30 to 40 wt % of the product slate, excluding catalyst coke, char, and vapors that are too large or polar to pass through the chromatography column. Further, continous time on stream data cannot be collected in the configuration described above.

#### Molecular Beam Mass Spectrometer (MBMS) Experiments

Catalyst deactivation studies were conducted using a fixed bed of catalyst with upgraded vapors continuously analyzed via online MBMS. In each experiment, 250 mg of catalyst was crushed and sieved to between 300 and 500 μm, held in place between plugs of quartz wool, and exposed to pyrolysis vapors generated by pyrolyzing 25 mg pine biomass every 3 min up to a biomass to catalyst ratio (B/C) of 3. B/C is measured on a solid biomass basis. Pyrolysis and catalytic upgrading were both conducted at 500 °C in a stream of 400 mL/min He (UHP, Matheson) and 5 mL/min Ar (UHP, Matheson) used as a tracer gas. Upgraded vapors were diluted in 3000 mL/min additional He to prevent further reactions and fed through a 250 μm sampling orifice where they were adiabatically expanded in a chamber at 100 mTorr. The expanded gas was formed into a molecular beam and ionized via electron impact ionization at 22.5 eV. Ions were measured using a quadrupole mass spectrometer scanning m/Z from 10 to 510 every second. The MBMS system allows for continuous exposure of a catalyst to pyrolysis vapors and produces enough spent catalyst for coke characterization. The MBMS also allows for observation of larger products than chromatography-based systems. However, the yield of observed products cannot be easily quantified. Instead, each product is described by its fraction of all observed ions.

#### Semi-Integrated 2" Fluidized Bed Reactor (2FBR) Experiments

Scheme 1 describes the important units within the 2FBR system. Biomass was fed at 300 g/h into a 2″ inner-diameter bubbling bed of 0.5 mm quartz sand held at 500 °C via a K-Tron loss-in-weight feeder connected to an auger. 17.6 sL/min nitrogen was used as a carrier and fluidizing gas. Solid char and vapors were separated in a cyclone with the vapors proceeding to a second bubbling bed where they were catalytically upgraded at 500 °C using formed catalysts sieved to above 300 μm. Fresh catalyst was continuously fed from a second K-Tron loss-in-weight feeder to the bubbling bed through an auger to maintain a desired B/C of 2.7. Catalyst was continuously removed from the upgrading reactor through an overflow tube to maintain a steady state catalyst load. Upgraded vapors were passed through a 2 μm stainless-steel mesh hot gas filter to remove catalyst fines before entering the condensation system which consisted of a jacketed condenser cooled to −2 °C by a chiller, an electrostatic precipitator, three dry ice-cooled condensation pots, and an ice-cooled coalescing filter. Gases exiting the condensation train were analyzed using an online moisture sensor, a dry test meter to measure volumetric flow rates, an online Agilent 490 micro-GC for H_2_ and C1–C4 quantification, a nondispersive infrared analyzer for CO, CO_2_, CH_4_, and O_2_ quantification (Model 300 from California Analytical Instruments), and an online Agilent 7890B GCMS system connected to a 5977A mass selective detector and a flame ionization detector for condensable gas quantification. All lines between the pyrolyzer and the condensation system were heated to between 400 and 500 °C using electric heat tapes.

**Scheme 1:**
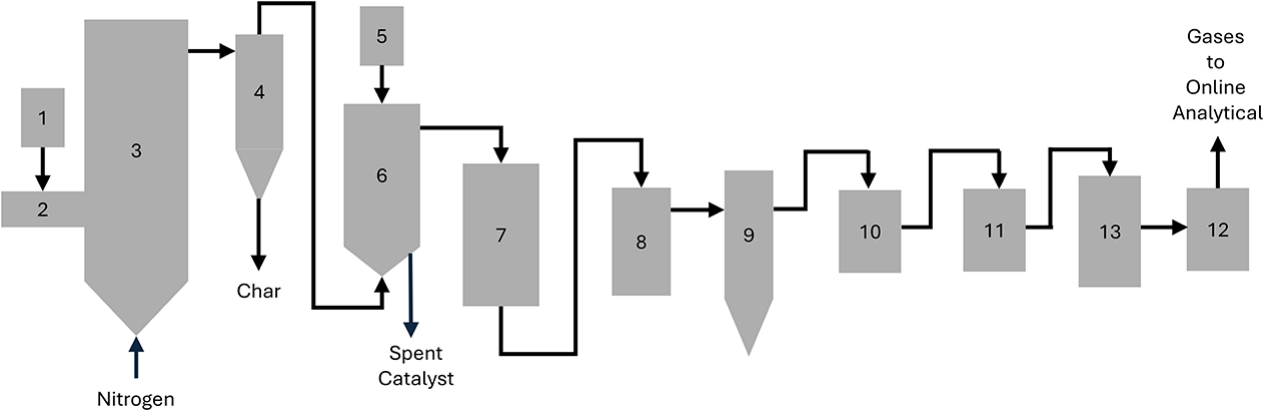
Schematic of the 2FBR System Showing the (1) Biomass Loss-in-Weight Feeder, (2) Biomass Feed Auger, (3) Fluidized Bed Pyrolysis Reactor, (4) Char Recovery Cyclone, (5) Catalyst Loss-in-Weight Feeder, (6) Fluidized Bed Catalytic Upgrading Reactor, (7) Hot Gas Filter, (8) Ethylene Glycol Cooled Condenser, (9) Electrostatic Precipitator, (10–12) Dry Ice-Cooled Condensers, and (13) Wet Ice-Cooled Coalescing Filter

All experiments fed a total of 1800 g biomass. Product liquids from the jacketed condenser, the electrostatic precipitator, and the dry ice condensers were phase separated into aqueous and bio-oil fractions. Both liquid phases were analyzed for CHN content in a LECO analyzer with O content assessed by difference, for S content using inductively coupled plasma-optical emission spectroscopy, and for water content using Karl Fischer titration. Chemical compositions of the oils were determined using a GCMS system equipped with a polyarc converter and a flame ionization detector. Relative standard deviations for detected compounds in a calibration mixture between experimental samples was less than 5%. The 2FBR system allows for recovery of liter quantities of bio-oil for downstream testing and analysis. However, it requires kilograms of formed catalyst for operation and therefore is only used with prescreened catalyst compositions established on the microreactor and MBMS systems.

#### Hydrotreating Experiments

CFP oils were hydrotreated in a continuous trickle-bed reactor described previously.^33^ Hydrotreating was conducted under 125 bar (1800 psig) H_2_ containing 60 ppm of H_2_S over a sulfided NiMo/Al_2_O_3_ catalyst (Johnson Matthey, 3.7 wt % Ni, 11.5 wt % Mo, 170 m^2^/g, 0.41 cm^3^/g).^33^ A mixture of 14 g catalyst and 28 g SiC (Panadyne) was placed in the nonisothermal zone, where the reactor heated from 100 °C to the operating temperature of 385 °C, and 12.5 g catalyst was placed in the isothermal operation zone at 385 °C. The catalyst was presulfided in a stream of 0.05 mL/min 35 wt % di*tert*-butyl disulfide in decane evaporated into the H_2_/H_2_S mixture at 150 °C for 2 h and then at 385 °C for 4 h. Bio-oil was fed via a 500 mL ISCO syringe pump at a rate of 2.5 mL/min to achieve a total weight space velocity of 0.1 g_oil_/g_cat_/h (0.2 g_oil_/g_cat_/h if only considering catalyst in the isothermal zone), and the H_2_/H_2_S mixture was fed at 175 mL/min for a ratio of 4,200 sL H_2_/L oil. Bio-oil produced with the ZSM-5 catalyst was first hydrotreated for 86 h followed by 73 h of hydrotreating for the P-ZSM-5 oil over the same catalyst bed. Liquid products were collected approximately every 12 h in a chilled 5 °C vessel followed by a −10 °C secondary vessel. Effluent gas flow rates were measured by a Coriolis flow meter and the composition of gaseous products was quantified using a micro-GC and a nondispersive infrared analyzer.

Product aqueous and organic phases were separated prior to characterization. Liquid organic products were analyzed for their elemental CHNS content, direct oxygen, and water by Karl Fischer titration. Fuel fractions were separated by distillation on a B/R 800 spinning band system equipped with a metal band and 14 theoretical plates. Distillation was first carried out at between 30 and 100 °C at atmospheric pressure. The remaining liquid was then allowed to cool, and the pressure was reduced to 30 Torr. All fractions were prepared and analyzed according to ASTM D2887.

### Catalyst Characterization

#### Inductively Coupled Plasma-Optical Emission Spectroscopy (ICP-OES)

Approximately 250 mg of sample was mineralized in a Teflon tube with 2 mL of concentrated HNO_3_, 1 mL of concentrated fluoroboric acid, and 5 mL of concentrated hydrochloric acid using a microwave digestion system (UltraWAVE 2, Milestone) at 1500 W following the UW-GE-4 method provided by Milestone. The sample was then diluted to 50 mL with distilled water with 0.25 mL of 1000 ppm yttrium in 2% HNO_3_ (Accustandard; New Haven, CT) added to serve as an internal standard for analysis by ICP-OES (ICP-OES 5100; Agilent Technologies Inc.).

#### Thermogravimetric Analysis (TGA)

Approximately 50 mg of sample was loaded into an Al_2_O_3_ sample pan and loaded into a Setaram Setsys Evolution TGA. The sample was first heated to 110 °C at 5 °C/min in flowing N_2_ to remove any adsorbed water. The gas composition was then changed to dry air (ZeroAir, Matheson) and the sample was heated to 700 °C at 10 °C/min while tracking the sample mass loss. The coke content of the sample was calculated by measuring the mass lost by the sample while in the stream of dry air.

#### NH_3_ Temperature-Programmed Desorption (TPD)

Acid site density measurements were performed using NH_3_ TPD on a microflow reactor system (Altamira Instruments) equipped with a thermal conductivity detector. Fresh catalysts (∼200 mg) were pretreated by heating in He (Ultrapure, Matheson) to 500 °C for 60 min, and then cooled to 120 °C in He flow. Next, NH_3_ adsorption consisted of flowing 10% NH_3_/He (Matheson) for 30 min at 120 °C, followed by flushing with He. The TPD was performed by heating at 30 °C/min from 120 to 500 °C, with a 30 min hold at 500 °C. The gas flow rate in all steps was 25 sccm. A sample loop of known volume (0.5 mL) was used to calibrate the thermal conductivity detector (TCD) response for NH_3_ to quantify the amount of NH_3_ desorbed from the catalysts. The amount of NH_3_ desorption was determined by integrating the area under the TCD response.

#### X-ray Diffraction (XRD)

XRD patterns were collected on catalyst samples using a Rigaku Ultima IV diffractometer with a Cu Kα X-ray source. Diffraction patterns were collected from 5 to 70° two-theta at a spacing of 0.04° and at a scan rate of 5° two-theta per minute with a dTex detector.

#### Nuclear Magnetic Resonance (NMR) Spectroscopy

Solid state magic angle spinning (MAS) NMR experiments were performed at 11.4 T (500 MHz) on a Bruker Avance III HD spectrometer, using a 3.2 mm MAS probe for ^27^Al NMR experiments at 10 kHz and 1.3 mm probe for ^31^P experiments at 50 kHz spinning speed. ^27^Al spectra were acquired with a rotor synchronized echo pulse sequence (90°-τ-180°-τ-acq), where τ = 1/νr. A π/2 pulse width of 1.6 μs was used with pulse recycle delays of 2 s. The ^27^Al spectra were referenced to 1 M Al­(NO_3_)_3_ at 0 ppm. ^31^P NMR experiments were acquired with a rotor synchronized echo pulse sequence (90°-τ-180°-τ-acq), where τ = 1/νr. A π/2 pulse width of 1.4 μs was used with pulse recycle delays of 2 s. The spectra were referenced to 85 wt % H_3_PO_4_ at 0 ppm.

#### Temperature-Programmed Oxidation (TPO)

TPO was conducted on a Micromeritics ASAP 2920 instrument. In each experiment, 40 mg of sample (<100 mesh) was loaded into the sample tube and dried at 120 °C for 10 min before ramping to 700 °C at 10 °C/min in 5 vol % O_2_ in He. The formation of CO and CO_2_ were monitored by online mass spectrometry calibrated using CO_2_ and CO cylinders.

#### Scanning Transmission Electron Microscopy with Energy Dispersive Spectroscopy (STEM-EDS)

The high resolution (HR)-STEM imaging and EDS was performed on an aberration-corrected JEOL *JEM-ARM200F NEOARM TEM/STEM* operated at 200 kV with a cold field emission gun (Cold-FEG), a next-generation Cs probe corrector (ASCOR) that compensates for higher order aberrations and energy dispersive X-ray spectroscopy (EDS) system with dual JEOL 100 mm^2^ silicon-drift detectors (SDD) for chemical analysis. STEM-EDS was acquired with a beam current of 94 pA for imaging and 423 pA for EDS with the data analyzed in the JEOL Analysis Station software. For STEM analysis of fresh materials, crushed catalysts were drop-cast onto lacey carbon-coated copper grids (SPI Supplies part no. Z3820C) from isopropanol suspensions. The specimens for STEM analysis of spent catalysts were prepared using a Hitachi NB5000 focused ion beam (FIB, Hitachi High Technologies Corp.) operated at 40 kV. Before FIB milling, a ∼1 μm thick protective carbon layer was deposited on the specimens to protect the surface of the area of interest during high-energy gallium (Ga) ion bombardment.

#### Raman Spectroscopy

UV Raman spectra of fresh ZSM-5 and P-ZSM-5 were collected at a 250 nm laser wavelength under in situ inert conditions following thermal pretreatment at 210 °C in air for 6 h. The laser excitation wavelength of 250 nm was provided by the third harmonic generation output of 750 nm laser line from a 4 kHz repetition rate, nanosecond pulsed, wavelength-tunable Ti:sapphire laser (Coherent, Indigo-S). A collimated laser light was focused onto the sample, and the scattered light from the sample refocused with a homemade 90° off-axis ellipsoidal reflector with the backscattering geometry onto the entrance slit of a triple-grating spectrometer (Princeton Instruments, Trivista 555) where Rayleigh light was filtered out and stray light significantly suppressed.^34^ The Raman light was collected by a liquid N_2_-cooled CCD detector (Princeton Instruments, SPEC-10). All Raman spectra of the samples were obtained in a homemade in situ Raman cell in flowing helium (∼100 mL/min) at room temperature.

Visible Raman spectra of post-CFP ZSM-5 and P-ZSM-5 samples were collected using a Horiba Scientific LabRam HR Evolution Raman Spectrometer. Spectra were measured using a single frequency 532 nm laser, a Horiba Scientific Synapse+ detector, and a 600 groove diffraction grating. Sample acquisition times and laser power were adjusted to provide optimal signal-to-noise ratios for the materials evaluated. To assess the nature and presence of surface carbon species following regeneration, the spent materials were further treated (ex situ) under 5% O_2_/He at 500 and 550 °C for 1 and 1.5 h, respectively, using a Micromeritics AutoChem II 2920 chemisorption analyzer.

#### Transmission Infrared (IR) Spectroscopy of Pyridine Adsorption

Pyridine-IR was collected using a Nicolet iS50 instrument (Thermo Scientific) at a resolution of 4 cm^–1^. The sample was pressed into a self-supported wafer before being loaded into the cell. The sample was evacuated followed by elevating the temperature to 450 °C for 1 h. The background was collected when the temperature was decreased to 150 °C. Pyridine was dosed into the sample cell at 0.1 mbar and allowed to adsorb for 30 min. The spectra of pyridine adsorption were collected after removing pysisorbed pyridine under evacuation (10^–6^ mbar) for 30 min. The Bro̷nsted-to-Lewis acid site ratio (*n*_Py‑B_/*n*_Py‑L_ ratio) is determined by the intensities of the bands at 1545 cm^–1^(Py-B) and 1445 cm^–1^(Py-L):

$$\frac{n_{\mathrm{Py}‐B}}{n_{\mathrm{Py}‐L}}=\frac{I_{\mathrm{Py}‐B,1545}\times \varepsilon_{\mathrm{Py}‐L,1445}}{I_{\mathrm{Py}‐L,1445}\times \varepsilon_{\mathrm{Py}‐B,1545}}$$ 1

where *I*_Py‑B,1545_ and *I*_Py‑L,1445_ represent the intensities of Py-B band at 1545 cm^–1^ and Py-L band at 1445 cm^–1^, respectively; ε_Py‑B,1545_ and ε_Py‑L,1445_ denote the extinction coefficients for these two bands. The extinction coefficient ratio was previously determined to be 1.33:^35,36^

$$\frac{\varepsilon_{\mathrm{Py}‐L,1445}}{\varepsilon_{\mathrm{Py}‐B,1545}}=1.33$$ 2

### Technoeconomic Analysis (TEA) and Greenhouse Gas (GHG) Emission Calculation

The main operations in the TEA model are shown in Scheme 2. The conceptual model was described in detail in a 2015 design report and assumes a plant capacity of 2,000 t of dry woody biomass per day.^37^ The same model framework was adapted for this analysis; key differences in the front-end CFP section compared to the 2015 report include a lower pressure of ∼2 bar absolute (vs ∼8 bar) and no H_2_ feed with the fluidizing gases. Cost estimates are reported in 2020 US dollars. A 50:50 blend of pine and forest residue feedstock was modeled at a cost of $72.53/dry US ton based on a later assessment.^38^ Other assumptions and methods are maintained from similar recent analysis reported by Griffin et al.^3^ with catalyst costs updated to $4.17/kg for ZSM-5 and with a 3.4% increase for P-ZSM-5, as estimated by the CatCost tool.^39^ Catalyst cost for ZSM-5 was estimated for a process synthesizing 450 tons of ZSM-5 catalyst containing 20 wt % Al_2_O_3_ binder based on a reported technique. All reagents, unit operations, utilities, catalyst disposal fees, and other costs are listed in Tables S3–S20. The synthesis cost of P-ZSM-5 was estimated by adding the necessary amount of phosphoric acid to the reagent list.

**Scheme 2:**
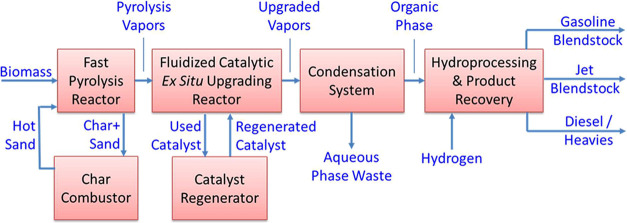
Simplified Block Flow Diagram of Ex Situ CFP Conceptual Process

Briefly, the conceptual process includes a fast pyrolysis reactor where biomass is converted to vapors, permanent gases, and char in a circulating dual fluid bed system with sand as the heating medium. After the fast pyrolysis reactions in an entrained flow reactor at ∼500 °C, the char and sand are separated using cyclones, and the char is combusted in the second fluidized bed to heat the sand, which is circulated back into the fast pyrolysis reactor to sustain the endothermic pyrolysis reactions. A sand replacement rate of 1.6% per day is used to remove attritted sand along with accumulated ash from the pyrolysis reactor system. The conceptual process uses an entrained flow system instead of a bubbling bed reactor used for the experiments because entrained flow systems offer improved control over residence time at larger scales and can achieve high throughputs.^40^ After solids are removed by cyclones, the vapors enter a similar dual fluid bed reactor at 500 °C with catalyst for upgrading the vapors in the first bed; after separation using cyclones, coke is burnt off the catalyst in the second bed and circulated back into the first bed. After catalytic upgrading the vapors and gases are sent through two absorber/condensers to condense the liquid. Gases are partially recycled to the fast pyrolysis reactor for fluidization of biomass, and the remainder of the gases used for H_2_ production in a steam reformer to meet all the H_2_ demand for hydrotreating and the remainder as fuel in the process and to produce steam for power generation. The liquid product separates into an aqueous phase and an organic liquid phase. The aqueous phase is sent to a regenerative thermal oxidizer to burn its organic content. The organic liquid from the two absorber/condensers are collected and sent for hydrotreating. The hydrotreated liquid is fractionated into gasoline, jet, and diesel cuts. Since the cuts may be altered depending on the most valuable target fuel blendstock, we report our cost estimates based on the overall energy content of the total liquid fuel product. In addition to these main steps, the model includes heat integration to use excess heat within the process for preheating streams to the extent feasible. Additional high-quality heat available is used for steam generation for electricity production. Excess electricity is exported to the grid. A minimum fuel selling price (MFSP) is estimated after accounting for all costs based on process model inputs and outputs.

GHG emissions were estimated using the lifecycle inventory (LCI) from the conversion process model. GHG emissions were estimated using Argonne National Laboratory’s “GREET-Based Interactive Life-Cycle Assessment of BETO Biofuel Pathyways” tool based on GREET 2023 rev1.^41^ Water use in the conversion process was also accounted for by the process model following the template used in our previous report.^37^

## Results and Discussion

Microscale screening experiments were first used to determine the optimal P loading in ZSM-5 for biomass CFP. Literature results show that as little as 0.1 wt % P can impact product slates for the methanol-to-olefins reaction,^24^ while it is also known that P can react with extraframework Al to form a separate AlPO_4_ phase at P/Al ratios of 0.5 and above.^42^ With this in mind, 0.5 mm formed ZSM-5 spheres with 20 wt % Al_2_O_3_ binder were modified by the addition of phosphoric acid using incipient wetness impregnation to achieve nominal P loadings of 1, 2.5, and 5 wt %. The resulting catalysts were characterized using NH_3_ TPD and NMR to understand the impact of P on the acidity of the catalysts and determine where P is located within the catalysts. The results of these characterizations are collected in Figure 1.

**Figure 1:**
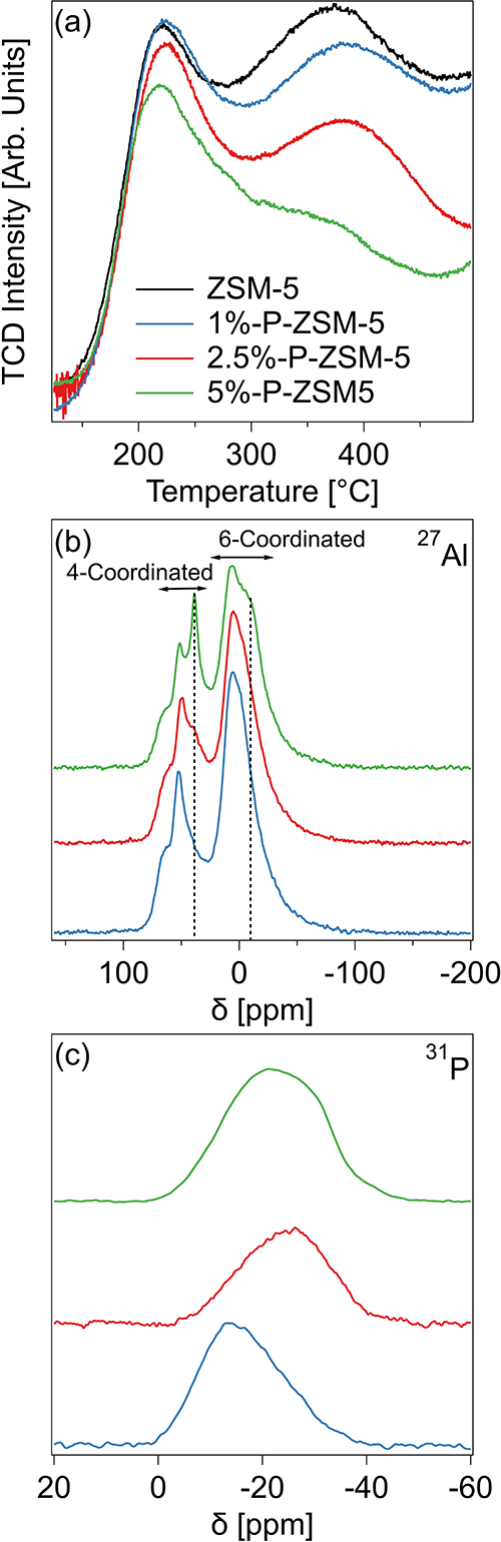
Characterization of P-ZSM-5 catalysts using (a) NH_3_ TPD, (b) ^27^Al MAS NMR, and (c) ^31^P MAS NMR.

NH_3_ TPD in Figure 1a shows that each catalyst displays a bimodal distribution of NH_3_ desorption temperatures centered at 225 and 400 °C. As the P loading increased, the high temperature peak was attenuated while the low temperature peak only decreased in intensity for 2.5%-P-ZSM-5 and 5%-P-ZSM-5, meaning that P preferentially interacts with strong acid sites. The addition of P to ZSM-5 led to a decrease in total ZSM-5 acid site density from 0.77 mmol NH_3_/g for fresh ZSM-5 to 0.29 mmol NH_3_/g for 5%-P-ZSM-5 (Table 1). The incorporation of P into the ZSM-5 catalyst was also studied using a combination of ^27^Al and ^31^P solid state MAS NMR (Figure 1b,c). In ^27^Al MAS NMR, Al species can be broadly separated into tetrahedrally coordinated species observed at chemical shifts between 40 and 70 ppm and octahedrally coordinated species between −10 and 30 ppm.^43^ The peak at 50 ppm is most commonly ascribed to tetrahedrally coordinated Al sites within the zeolite framework while the shoulder at 60 ppm corresponds to disordered tetrahedral Al species either in the zeolite or in amorphous Al_2_O_3._ ^44^ Similarly, the peak centered at 0 ppm is ascribed to octahedrally coordinated Al in Al_2_O_3_. All these peaks are observed in the ^27^Al NMR of the unmodified ZSM-5 in Figure S2. As the P concentration increases, new peaks at 45 and −5 ppm increase in intensity and are ascribed to tetrahedral and octahedral Al species coordinated to P respectively.^43^ This result indicates that P interacts both with the Al in the zeolite framework and in the Al_2_O_3_ binder. As the P loading increased, the intensity of the peak at 50 ppm decreased relative to the peak centered at 0 ppm potentially indicating some dealumination of the zeolite which may have occurred due to hydrothermal conditions during calcination of the phosphoric acid impregnated ZSM-5 catalyst.^23,28^ However, the presence of Al in the catalyst binder makes quantitative statements about the state of zeolite Al difficult.

**Table 1:** Properties of 1-, 2.5-, and 5%-P-ZSM-5 Catalysts Compared to Unmodified ZSM-5^a^

	ZSM-5	1 wt % P-ZSM-5	2.5 wt % P-ZSM-5	5 wt % P-ZSM-5
P loading (wt %)		1.16	2.71	4.21
surface area (m^2^/g)	389	325	322	297
acid site density (mmol/g)	0.77	0.46	0.34	0.29

a P loading was determined by ICP-OES, and measurement data for N_2_ physisorption surface area and XRD crystallite size calculations can be found in Figures S3 and S4, respectively.

^31^P MAS NMR in Figure 1c shows a single broad resonance between −40 and 0 ppm. The lack of a single resolved peak suggests that P does not exist as well-defined species but instead occupies a broad range of disordered states. Previous studies have ascribed higher chemical shift peaks near −15 ppm to P associated with ZSM-5 framework Al while peaks with lower shifts are ascribed to the formation of polyphosphates associated with Al.^45^ As the P loading increases from 1%-P-ZSM-5 to 5%-P-ZSM-5, the ^31^P NMR signal shifts to lower resonances, potentially indicating increased polyphosphate formation as available Al binding sites are occupied.

Analysis of N_2_ physisorption results in Figure S3 indicate that the addition of P led to a decrease in the overall surface area from 389 m^2^/g for the fresh ZSM-5 to 297 m^2^/g for 5%-P-ZSM-5 (Table 1). These findings align with previous results showing that the P reduces the acidity of zeolite catalysts and leads to pore occlusion.^30^ The addition of P led to a decrease in the average ZSM-5 crystallite size, as determined by Scherrer analysis of the XRD patters, from 41.9 nm in the unmodified ZSM-5 to around 34 nm for the three P-modified materials (Figure S4). This indicates a perturbation of the ZSM-5 crystalline structure, though there was no clear trend of decreasing crystallinity with increasing P loading. This disruption in the crystalline structure may also have contributed to the loss of surface area observed by N_2_ physisorption. Tabulated acidic, elemental, and textural data are collected in Table 1 and suggest that increasing the loading of P increases the amount of interaction between P and ZSM-5 framework Al responsible for its strong acidity. This conclusion is supported by the loss of acid site density in NH_3_ TPD and the decrease in tetrahedral Al signal in ^27^Al NMR. However, P does not interact solely with zeolite Al and likely reacts with Al in the Al_2_O_3_ binder as shown by the presence of octahedral Al–P species in the ^27^Al MAS NMR. Finally, higher P loading also leads to increasing formation of polyphosphate groups as shown by the ^31^P MAS NMR.

The catalysts were crushed into powders and evaluated for their initial performance in biomass ex situ CFP in a microreactor system at 500 °C. Biomass was pyrolyzed in 1 mg batches and the resulting vapor pulses were upgraded over a 5 mg fixed bed of catalyst to achieve a biomass to catalyst ratio (B/C) of 0.2. The resulting vapors were fed directly into a GCMS-FID system for characterization and quantification. It is important to note that the GCMS-FID system can only characterize 30 to 40 wt % of the product slate, excluding catalyst coke, char, and vapors that are too large or polar to pass through the chromatography column.

Figure 2 shows the yield of molecules containing at least 6 carbons, which are expected to exit hydrotreating in the fuel range, as well as the yield of oxygenate molecules as a function of P loading. The yield of C6+ molecules in Figure 2a increases for 1%-P-ZSM-5 and 2.5%-P-ZSM-5 relative to the baseline ZSM-5 catalyst, while 5%-P-ZSM-5 produces a similar C6+ yield to ZSM-5. However, only the yield of C6+ products for 2.5%-P-ZSM-5 is above the baseline catalyst when considering experimental uncertainty. As P loading increases, Figure 2b shows that the oxygenated molecule yield also increases, with 2.5%-P-ZSM-5 and 5%-P-ZSM-5 producing significantly more oxygenates than the baseline ZSM-5. This is likely due to the loss of acid sites and changes to the zeolite structure and accessibility from P modification, as discussed above. The results in Table 1 suggest that the loss in acid site density is driven more by P interaction with the acid sites themselves, than pore disruption. For example, the 5%-P-ZSM-5 had 62% fewer acid sites than the unmodified ZSM-5 but only lost 23% of its surface area relative to the unmodified material. The strongest Bro̷nsted acid sites, which are most impacted by P addition according to NH_3_ TPD, are responsible both for deoxygenation and cracking reactions,^46^ as well as hydrogen transfer reactions that promote coke formation.^47^ Therefore, higher P loadings may produce fewer C6+ molecules due to a lack of sufficient cracking activity caused by acid site loss.

**Figure 2:**
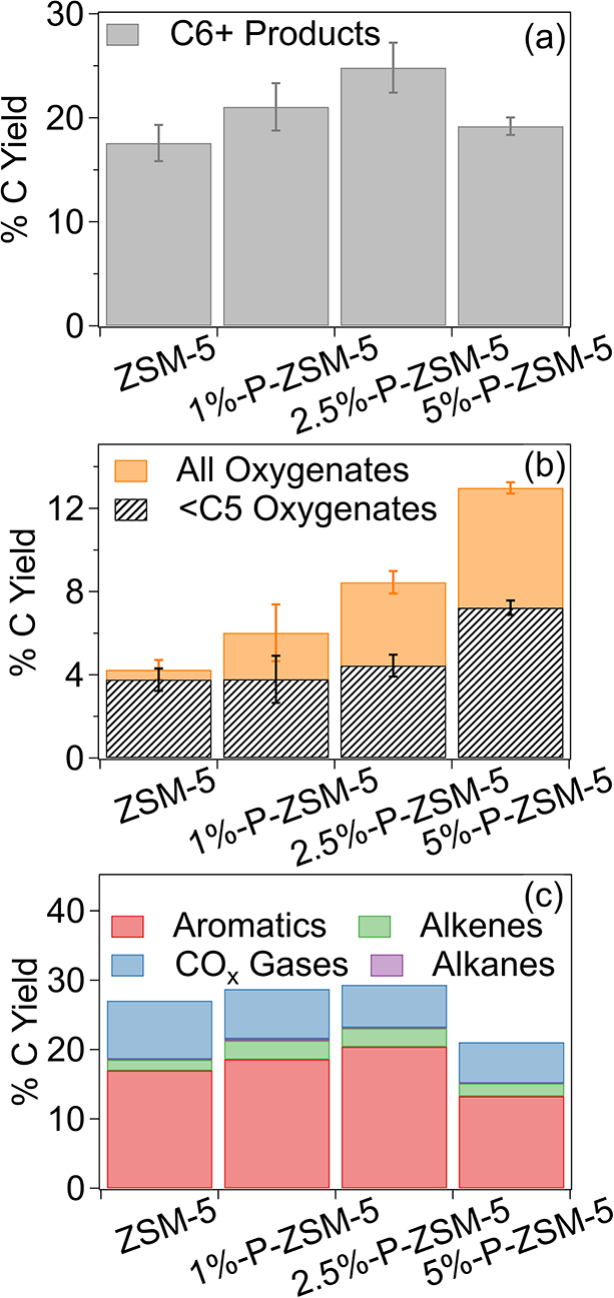
Carbon yield to (a) products with at least six carbons and (b) oxygenated molecules and (c) aromatic, alkane, alkene, and CO_*x*_ gases for ex situ microreactor CFP experiments run at 500 °C and a B/C of 0.2. Error bars represent the standard deviation of five replicate experiments.

Figure 2b shows that higher P loading corresponds to an increase in formation of light oxygenate with fewer than 5 carbons. These light oxygenates, especially short ketones and acids, contribute to bio-oil instability,^5,48,49^ and are unlikely to be upgraded into the fuel range. Complete product distributions observed for each catalyst are available in Figure 2c. Notably, both 1%-P-ZSM-5 and 2.5%-P-ZSM-5 produced more aromatic products and fewer CO_*x*_ gases (CO and CO_2_) than ZSM-5. 5%-P-ZSM-5 produced fewer aromatics and CO_*x*_ gases than the baseline ZSM-5. We hypothesize that decreasing acid site density of ZSM-5 with P addition reduces overcracking to light gases and hydrogen transfer reactions which consume aromatic molecules to form coke.^23^ These benefits are eventually outweighed by a loss of catalytic acid sites in 5%-P-ZSM-5 both through binding with P and through possible occlusion by polyphosphates. The increase in C6+ oxygenates in Figure 2b and aromatics in Figure 2c going from unmodified ZSM-5 to 2.5%-P-ZSM-5 likely both contribute to the overall increase in C6+ products observed in Figure 2a. Based on their initial product slates, the 1%-P-ZSM-5 and 2.5%-P-ZSM-5 catalysts were selected for further investigation in a semicontinuous process.

1%-P-ZSM-5 and 2.5%-P-ZSM-5 were compared to the baseline ZSM-5 in a pyrolysis reactor connected to an MBMS for detection of a broader set of product molecules as a function of time on stream. This system enabled continuous monitoring of catalyst performance to compare their deactivation and regeneration behavior and generated sufficient postreaction catalyst samples for thermogravimetric analysis. Each catalyst was crushed and sieved to a particle size between 300 and 500 μm and exposed to pulses of biomass pyrolysis vapors up to a B/C of 3, as this B/C has been demonstrated to cause significant catalyst deactivation via coking.^17,50^ To classify products, selected mass fragments were assigned to either hydrocarbon or raw pyrolysis vapor categories based on previous work.^17^ Table S2 lists the specific masses tracked in each product category as well as their molecular assignments. Hydrocarbons of interest are comprised of light olefin and aromatic fragments while raw pyrolysis vapors are represented by several common fragments for aldehydes and furans as well as fragments observed during noncatalytic pyrolysis of pine feedstocks.^17^ Figure 3a tracks the fraction of all ions classified as hydrocarbons, as a function of increasing B/C, while Figure 3b represents the fraction of total ions classified as raw pyrolysis vapors. Figure 3a shows that both 1%-P-ZSM-5 and 2.5%-P-ZSM-5 initially produce a lower fraction of hydrocarbons than the unmodified ZSM-5. This can be explained by their lower total acidity, known to be responsible for deoxygenation reactions during CFP.^10^ However, as B/C increases, the unmodified ZSM-5 loses hydrocarbon selectivity more quickly than the P-modified catalysts, with 2.5%-P-ZSM-5 showing the highest hydrocarbon selectivity at a B/C of 3 while 1%-P-ZSM-5 hydrocarbon selectivity converged to that of the unmodified catalyst.

**Figure 3:**
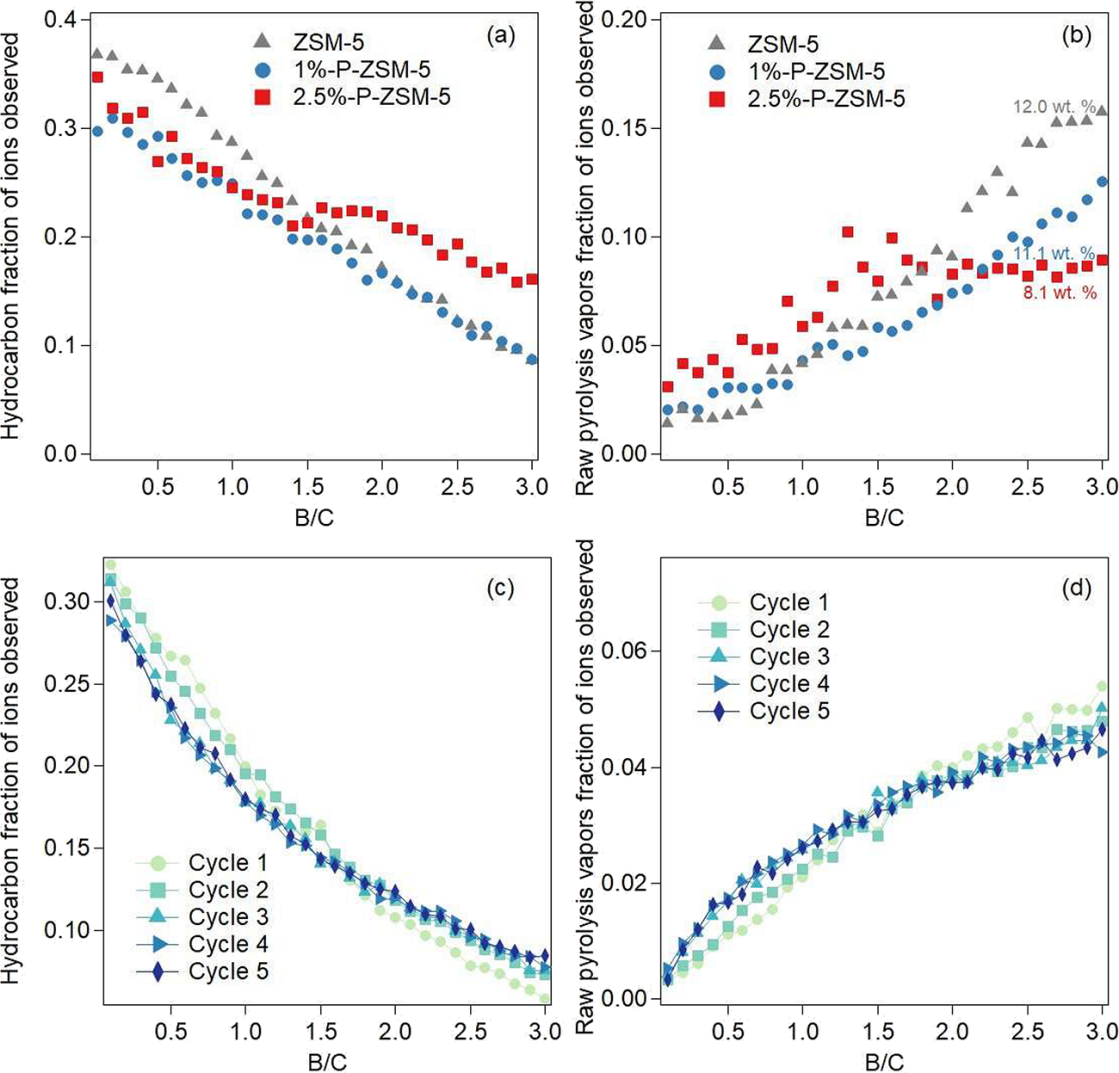
Fraction of all MBMS ions observed corresponding to (a,c) hydrocarbons, and (b,d) raw pyrolysis vapors for (a,b) fresh catalysts and (c,d) 2.5%-P-ZSM-5 used for five consecutive cycles as a function of B/C. Ion masses tracked for each fraction are described in Table S2. Final catalyst coke contents are labeled in (b). All catalysts were tested at 500 °C. Lines in c,d are used to better identify each data set.

The trends observed in hydrocarbon product selectivity aligned with the breakthrough of raw pyrolysis vapors as a function of B/C, as shown in Figure 3b. Initially, the P-modified catalysts produce more raw pyrolysis vapors than the unmodified ZSM-5, especially 2.5%-P-ZSM-5, consistent with its lower acidity and greater oxygenate productivity observed in Figure 2b. The selectivity for raw pyrolysis vapors increases for all catalysts as a function of time on stream. Zeolite deoxygenation activity typically decreases due to a combination of coke deposition and dealumination under hydrothermal conditions.^17,31^ However, both P-modified catalysts eventually produced fewer raw pyrolysis vapors than unmodified ZSM-5, indicating prolonged upgrading activity. At a B/C of 2, 1%-P-ZSM-5 surpasses 2.5%-P-ZSM-5 in raw pyrolysis vapor selectivity, suggesting that higher P loadings are preferable at longer times on stream. At a B/C of 3, unmodified ZSM-5 exhibited the greatest raw pyrolysis vapor ion fraction, 2.5%-P-ZSM-5 produced the fewest raw pyrolysis vapors, and 1%-P-ZSM-5 produced an intermediate selectivity. This selectivity trend mirrors that for fuel-range molecules in Figure 2a. The lower fraction of raw pyrolysis vapors over the P-modified catalysts in Figure 3b can be explained by less coke deposition onto the P-modified catalysts, allowing them to maintain access to their acid active sites needed for upgrading raw pyrolysis vapors. This hypothesis is bolstered by elemental analysis which shows that unmodified ZSM-5 accumulated 12.0 wt % coke during vapor upgrading while 1%-P-ZSM-5 and 2.5%-P-ZSM-5 accumulated 11.1 and 8.1 wt % coke, respectively. The decreasing coke content of the catalysts with increasing P loading corresponds to their improved raw pyrolysis vapor upgrading performance as a function of time on stream and can be ascribed to their lower strong acid site density which catalyzes coke-forming hydrogen transfer reactions.

Following the biomass pyrolysis vapor upgrading experiment in Figure 3a,b, the 2.5%-P-ZSM-5 catalyst was regenerated under oxidizing conditions and exposed to an identical biomass CFP reaction cycle. This process was repeated four times, providing catalyst performance data for five reaction cycles, which is collected in Figure 3c,d. Figure S5 compares the hydrocarbon and raw pyrolysis vapor ion fractions for each regeneration cycle to the initial catalyst activity at B/C values of 0.1, 1.5, and 3.0. At B/C 0.1 and 1.5, the catalyst shows less than 10% lower hydrocarbon selectivity than the initial catalytic cycle. However, the loss in selectivity does not increase with added cycles, possibly indicating an equilibration of the catalyst. Similarly, the catalyst shows increasing selectivity to hydrocarbons at higher cycle numbers, possibly due to slower deactivation of the equilibrated catalyst. The changes in raw pyrolysis vapor selectivity were smaller, with a decrease in raw pyrolysis vapors at a B/C of 3.0 representing the largest magnitude change. The complete restoration of activity following oxidation suggests that 2.5%-P-ZSM-5 was deactivated primarily by carbonaceous coke, with minimal impact from irreversible dealumination. These results align with previous observations for unmodified ZSM-5 regeneration.^3,18^ While five catalyst regeneration cycles are helpful for understanding the short-term regenerability of the 2.5%-P-ZSM-5 catalyst, longer term regeneration studies are needed to assess the full catalyst lifetime.

Based on its higher hydrocarbon and lower raw pyrolysis vapor production over the full operating cycle as well as its regenerability, a P loading of 2.5 wt % was selected for a large-scale (3.5 kg) synthesis of P-ZSM-5 denoted L2.5%-P-ZSM-5. This modification method is easily scalable as it avoids the use of organic solvents or other expensive reagents. A cost analysis was conducted using CatCost,^39^ a tool designed to estimate materials and process costs of large-scale catalyst production. This preliminary analysis indicated that at a production scale of 450 tons of catalyst, the required phosphoric acid increased the catalyst cost by 0.11 $/kg, or 2.5%, relative to the baseline ZSM-5. Collected model outputs for the baseline ZSM-5 catalyst and P-ZSM-5 can be found in Tables S3–S11 and S12–S20, respectively.

Table 2 compares the properties of L2.5%-P-ZSM-5 with the baseline formed ZSM-5. As observed previously, the addition of P decreased surface area from 389 m^2^/g to 312 m^2^/g due to partial pore occlusion and structural disruption. NH_3_ TPD (Figure S6) showed a decrease in acid site density from 1.06 to 0.85 mmol/g, with most of the loss accounted for by high temperature NH_3_ desorption, indicating a reduction in strong acidity. The Bro̷nsted-to-Lewis acid site ratio listed in Table 2 was calculated based on pyridine IR spectra in Figure S7 and was only slightly increased from 1.5 to 1.7 by the addition of P, suggesting minimal preference for P binding to Bro̷nsted acid sites. It is important to note that this modified catalyst was prepared using commercial formed spheres containing 20 wt % Al_2_O_3_ binder. Therefore, any changes observed by NH_3_ TPD, NMR, or IR include changes occurring at both zeolite and binder acid sites. It is reported in the literature that introducing phosphorus could lead to a decrease in the Lewis acid sites of the binder while it could also reduce the number of Bro̷nsted acid sites in ZSM-5.^51^ Finally, P addition reduced the ZSM-5 crystallite size observed by XRD from 39.3 to 25.8 nm. Similar results have been observed in the literature and are attributed to dealumination during P addition or the ensuing heat treatment.^23,28^ XRD does not indicate the presence of any new crystalline phases upon the addition of P.

**Table 2:** Characterization Data for ZSM-5 and L2.5%-P-ZSM-5^a^

	ZSM-5	L2.5%-P-ZSM-5
surface area (m^2^/g)	389	312
acid site density (mmol/g)	1.06	0.85
Bro̷nsted/Lewis acid site ratio	1.5	1.7
average crystallite size (nm)	39.3	25.8

a NH_3_ TPD, pyridine-IR, XRD, and N_2_ physisorption data are collected in Figures S6–S9.

The inclusion of P was confirmed using UV Raman spectroscopy and STEM-EDS mapping, shown in Figure 4a,b–l, respectively. UV Raman spectroscopy revealed similar Si–O–Si and Si–OH bands between 300 and 500 cm^–1^ and 800–840 cm^–1^ respectively for both L2.5%-P-ZSM-5 and ZSM-5.^52^ The addition of P introduced a new peak at approximately 1100 cm^–1^, attributed to P–O vibrations in [PO_4_]^3–^ groups interacting with Al, supporting previous observations that P binds to Al in these materials.^53^ STEM-EDS confirmed P incorporation and demonstrates that P is distributed throughout the catalyst. Notably, the P distribution Figure 4l resembles that of Al in Figure 4j, suggesting that P may favorably colocate with Al in the Al_2_O_3_ binder.^31^P MAS NMR analysis in Figure S10 indicates species similar to those observed in Figure 1c for the small-scale P-ZSM-5 catalyst synthesis. Specifically, the asymmetric peak shape in Figure S10 indicates that P exists in a distribution of disordered structures with a variety of coordinations to Al both in the ZSM-5 and in the Al_2_O_3_ binder.^45^ These characterization results confirm that P is incorporated into the formed catalyst but suggest P is distributed between the ZSM-5 and the catalyst binder.

**Figure 4:**
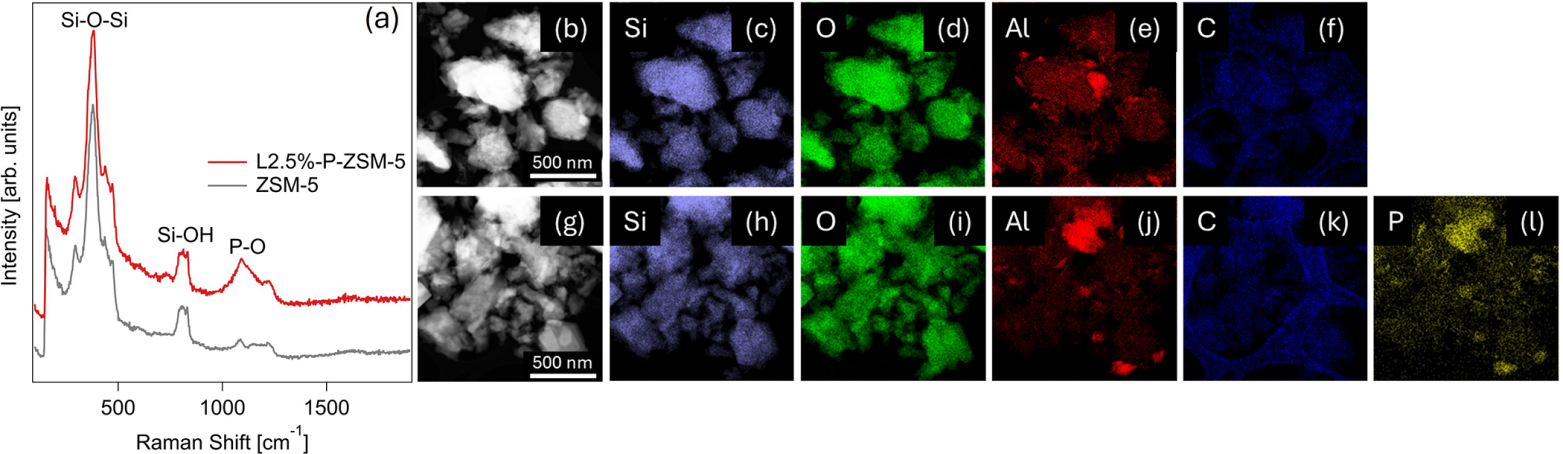
Characterization of P in L2.5%-P-ZSM-5 and baseline ZSM-5 using (a) UV Raman spectroscopy, and (b–l) STEM-EDS mapping of (c,h) Si, (d,i) O, (e,j) Al, (f,k) C, and (l) P.

The P-modified and baseline ZSM-5 catalysts were compared in an ex situ bubbling bed fluidized bed reactor system described elsewhere,^15^ where liquid products could be generated in liter quantities. A pine feedstock, containing 1.5 wt % ash and milled to below 0.5 mm was pyrolyzed at 500 °C in a bubbling bed of quartz sand. Pyrolysis vapors were upgraded over each catalyst at 500 °C and a B/C of 2.75. Both catalysts were tested in triplicate. Carbon balances for each material exceeded 90% and their distributions are shown in Figure 5 where error bars represent the standard deviation across three runs. Carbon and mass yields for the data in Figure 5 are summarized in Tables S21 and S22, respectively. Both catalysts produced similar char yields, 20 ± 2 C % for the unmodified ZSM-5 and 20.7 ± 0.7 C % for L2.5%-P-ZSM-5, consistent with char generation occurring prior to contact with the catalyst. Likewise, aqueous product carbon yields were similar, 6.1 ± 0.3 C % and 5.7 ± 0.4C % for the unmodified ZSM-5 and L2.5%-P-ZSM-5, respectively. Condensable gas yields, defined as molecules that bypassed the condensation system but are liquid at room temperature such as acetaldehyde, furans, and single-ring aromatics, were also similar at 7 ± 1 C % for the unmodified ZSM-5 and 7.2 ± 0.4 C % for the L2.5%-P-ZSM-5. These similarities underscore the comparable slate of molecules being produced by the two catalysts, which both operate through acid active sites.

**Figure 5:**
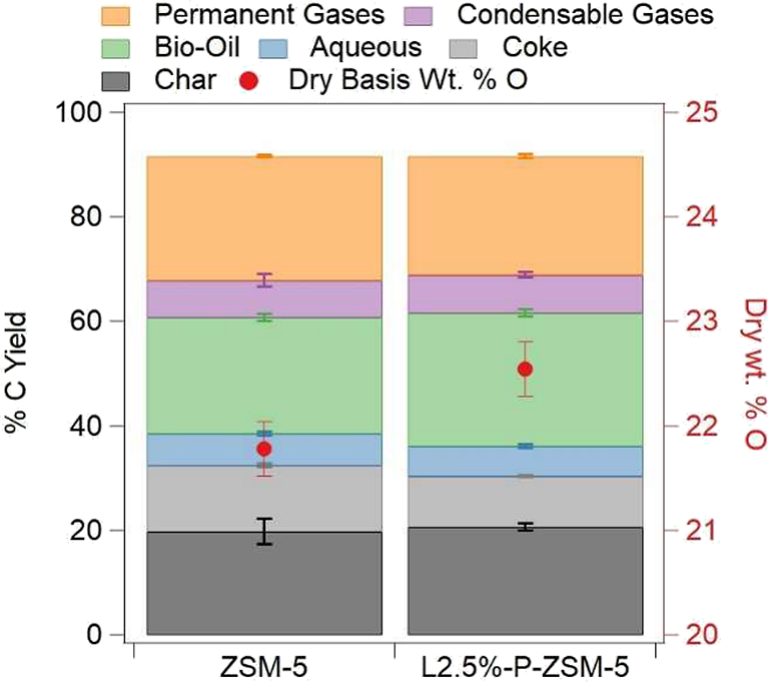
Carbon yield of each product fraction for unmodified ZSM-5 and L2.5%-P-ZSM-5 and the O content of the produced bio-oils. Numerical C yield results are collected in Table S21 and mass yield data are shown in Table S22.

The two catalysts differed in their selectivities to coke, bio-oil, and permanent gases. Most notably, L2.5%-P-ZSM-5 produced significantly less coke (9.7 ± 0.2 C %) than unmodified ZSM-5 (12.6 ± 0.4 C %) as well as recent ZSM-5 literature reports (13–15 C %).^3,4^ This aligns with the small-scale MBMS results and literature where the addition of P disrupts strong Bro̷nsted acid sites responsible for coke deposition.^23,25,26^ The majority of the carbon directed away from coke likely entered the bio-oil fraction, where the addition of P increased the carbon yield from 22.3 ± 0.7 C % for unmodified ZSM-5 to 25.6 ± 0.7 C % for L2.5%-P-ZSM-5. This increase is substantial relative to the baseline ZSM-5 experiment but falls within the range of recent pine CFP ZSM-5 results that observed bio-oil C yields between 21 and 28%.^3,4^ Increased bio-oil yield suggests that P modifies catalyst acidity reducing pathways that convert bio-oil range molecules to coke. Additionally, the bio-oil yield was further improved over L2.5%-P-ZSM-5 by a decrease in selectivity to permanent gases which dropped from 23.8 ± 0.2 C % over the unmodified catalyst to 22.7 ± 0.3 C % over L2.5%-P-ZSM-5. Table S23 provides speciation of the permanent gases and shows similar CO production over each catalyst while L2.5%-P-ZSM-5 produced less CO_2_, and fewer hydrocarbon gases than the unmodified ZSM-5. The decrease in permanent gases can be accounted for by the lower acid site density in L2.5%-P-ZSM-5, limiting overcracking of fuel-range molecules into light gases.^3^ However, this decrease in light gas production also resulted in less oxygen being removed from the pyrolysis vapors as CO_*x*_ gases, resulting in a higher dry oxygen content (22.5 ± 0.2 wt % O) for the L2.5%-P-ZSM-5 bio-oil relative to the unmodified ZSM-5 bio-oil (21.7 ± 0.2 wt % O). The lower oxygen content in the ZSM-5 bio-oil is not responsible for the increased coke production relative to L2.5%-P-ZSM-5 as previous studies have shown that the coke selectivity of unmodified ZSM-5 does not decline below 12 C % as B/C increases, and therefore oil oxygen content increases, beyond a B/C of 2.^54^ The increase in oil oxygen content is also a result of decreased catalyst acidity, which has been identified as the active site for many deoxygenation reactions.^10^ High oil oxygen content necessitates additional hydrogen in the hydrotreating step to produce a hydrocarbon fuel and could result in additional oil instability.^5^ While carbon balances are not closed to 100%, the difference in bio-oil yield between the two catalysts is outside of the experimental uncertainty from the triplicate measurements for each catalyst. Furthermore, the corresponding change in coke production is also outside of experimental uncertainty, confirming the statistical significance of the change in product slate between the two catalysts.

Post-reaction catalysts were compared using TPO and visible Raman spectroscopy to characterize deposited coke species in Figure 6. TPO results in Figure 6a,b indicate that P addition not only reduces coking rates but also changes the properties of the coke relative to unmodified ZSM-5. Analysis of the gases produced during TPO show that oxidation of L2.5%-P-ZSM-5 coke produced a lower CO_2_ to CO ratio (1.4) relative to unmodified ZSM-5 coke (3.0), suggesting differences in coke combustion behavior on the two catalysts. Previous studies have shown that catalyst composition affects coke structure, leading to variations in coke oxidation rates and products.^55,56^

**Figure 6:**
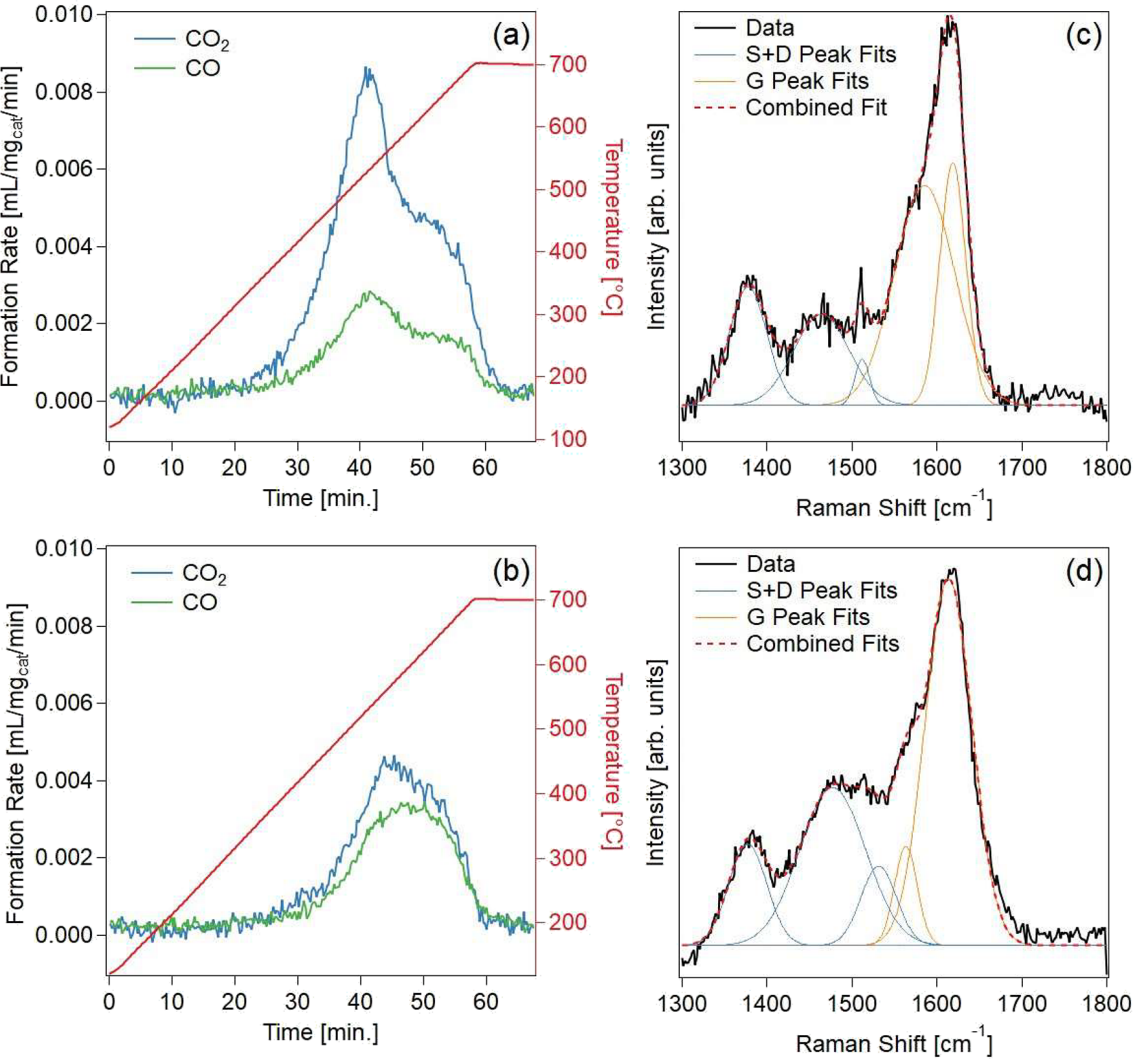
Coke characterization on ZSM-5 and L2.5%-P-ZSM-5 catalysts using (a,b) temperature-programmed oxidation and (c,d) visible Raman spectroscopy. S and D Raman bands are distinguished from the G band in (c,d) using Gaussian peak fits integrated in Table S6.

Visible Raman spectroscopy can be used to characterize the molecular structure of coke, and spectra for catalysts after CFP are collected in Figure 6c,d. Raman spectroscopy can distinguish between graphitic coke in the G band, centered at 1600 cm^–1^_,_ from disordered aromatic and alkyl coke in the S and D bands at 1300–1500 cm^–1^.^57,58^ Raman spectra in Figure 6c,d indicate that both catalysts contained both coke types. However, integration of the three peaks reveals that L2.5%-P-ZSM-5 contained a lower relative proportion of graphitic, G band coke (53%) compared to the unmodified ZSM-5 (65%). This could suggest that the coke in L2.5%-P-ZSM-5 is more disordered than that of ZSM-5, which may explain the comparatively low temperature oxidation in Figure 6a,b. Integrated Raman peak areas are collected in Table S24.

Finally, STEM-EDS was used to investigate the coke distribution in the spent ZSM-5 and 2.5%-P-ZSM-5 catalysts after 5 CFP cycles in the MBMS. Figure S11 and Table S25 show cross sectional elemental maps and corresponding mass fractions of elements detected by STEM-EDS highlighting the higher coke concentration in the unmodified ZSM-5 catalyst as well as the greater prevalence of carbon associated with the Al_2_O_3_ binder in the unmodified catalyst. Post reaction ^31^P MAS NMR in Figure S10 indicates that the distribution of local environments of P in L2.5%-P-ZSM-5 is not substantially changed by one cycle of CFP.

To evaluate the suitability of the bio-oils for downstream processing to fuels, the bio-oils produced by L2.5%-P-ZSM-5 and unmodified ZSM-5 catalysts were hydrotreated in a continuous bench-scale hydrotreater described previously.^4^ The hydrotreater used a sulfided NiMo/Al_2_O_3_ catalyst and operated at 385 °C under 125 bar H_2_ containing 60 ppm of H_2_S with a bio-oil feed rate of 0.2 g_oil_/g_catalyst_/h. The hydrotreated products were distilled into gasoline (below 110 °C), jet (110–275 °C), diesel (275–330 °C), and residue (above 330 °C) fractions based on their boiling points, as described in Table S26. The cutoff between fuel fractions were determined by taking many distillation cuts and measuring the fuel properties and analyzing them by ASTM D2887 simulated distillation. Fractions were then blended to maximize the aviation fuel yield and the blends were confirmed to conform to specifications of ASTM D2887 and D86. The yield of each liquid product from each hydrotreated oil, as well as the overall yield of fuels from biomass on a carbon basis, is shown in Table 3. The mass fraction of oil, aqueous products, and permanent gases from the hydrotreating step did not vary significantly between the two bio-oils. The jet fraction mass yields from bio-oil hydrotreating were 60% for ZSM-5 and 54% for L2.5%-P-ZSM-5, higher than recent experiments which reported 46–50% mass yield from CFP oil to jet fuel in the same hydrotreating reactor under similar operating conditions.^3,59^ However, the jet fuel yield depends on how distillation cuts are assigned and CFP oil properties. The results of this experiment are therefore most valuable for directly comparing fuel yields for ZSM-5 and L2.5%-P-ZSM-5 which were hydrotreated consecutively under identical conditions. Despite lower selectivity to jet fuel during hydrotreating relative to unmodified ZSM-5 bio-oil, the hydrotreated product from L2.5%-P-ZSM-5 bio-oil achieved an 11% greater carbon yield to jet fuel from biomass due to improved efficiency in the CFP step. Similarly, the addition of P increased the overall process fuel carbon yield, including gasoline, diesel, and jet, from 18.6 to 20.4%. The jet fuel fraction from each hydrotreated product was tested for its fuel properties according to ASTM 4054 with the results collected in Table S27. For the measured parameters, both jet fractions met nearly all ASTM specifications, each exhibiting flash points and lower heating values less than 10% below the specified range. Such minor fuel property shortcomings could be addressed by blending with other fuel streams or adjusting distillation conditions for isolating the jet fraction.

**Table 3:** Collected Phase Yield, Product Fractionation, and Carbon Efficiencies for CFP Bio-Oils Produced by ZSM-5 and L2.5%-P-ZSM-5 Catalysts^a^

	ZSM-5	L2.5%-P-ZSM-5
mass yields (g/g_CFP oil_)		
oil	0.66 ± 0.02	0.67 ± 0.01
aqueous	0.28 ± 0.003	0.29 ± 0.003
gases	0.05 ± 0.01	0.04 ± 0.003
distillation cuts (mass %)		
gasoline (below 110 °C)	21	19
jet (110–275 °C)	60	54
diesel (275–330 °C)	12	12
residue (above 330 °C)	6	9
losses	2	5
process C efficiencies (%)		
biomass to bio-oil	23	27
bio-oil to hydrotreated products	87	89
hydrotreated products to jet	60	54
overall biomass to jet C yield	12.0	13.0
overall biomass to fuel C yield	18.6	20.4
overall biomass to hydrotreated products C yield	19.8	22.6

a Uncertainties in the mass yields reflect the standard deviation of triplicate measurements.

The experimental data in Table 3 were used to modify the CFP and hydrotreating product slates in a technoeconomic and lifecycle assessment model described previously,^37^ that describes a 2000 dry ton per day facility including a circulating sand fluid bed fast pyrolysis reactor system followed by a similarly configured ex situ catalytic circulating fluid bed vapor upgrading system, condensation and separation of products, hydrotreating to fuels under the conditions used in this study, and associated utilities. Previous sensitivity analysis with this model identified CFP carbon yield to bio-oil as one of the key drivers impacting the ultimate fuel selling price.^37^ The models revealed that the addition of P to ZSM-5 resulted in a decrease in the minimum fuel selling price (MFSP) of the total fuel product on a gallon of gasoline energy equivalent (GGE) basis from 6.81 $/GGE to 5.86 $/GGE, a 14% decrease with a 19% increase in modeled yield; note that the TEA includes the energy value associated with residues within the product slate as they can be further hydrocracked to fuel products. The MFSP for the unmodified ZSM-5 catalyst is similar to a previous report using the same model and catalyst with similar performance, which estimated an MFSP of 6.63 $/GGE (adjusting to 2020 dollars),^3^ while the P-modified catalyst represents a substantial improvement. A lower MFSP was calculated with a ZSM-5 catalyst in another TEA for the same process (5.67 $/GGE in 2020 dollars), the difference is primarily due to a higher modeled CFP pressure of 8 bar which reduced reactor sizes and capital costs.^12,37^ Higher pressure experiments were not considered due to experimental safety considerations. Figure 7 illustrates the contribution of major process steps to the final fuel selling price. The most notable cost reductions in Figure 7 are in the feedstock and catalytic pyrolysis steps, which are also the largest cost drivers of the overall process. The decrease in cost when using P-ZSM-5 is driven largely by the increased efficiency of the process from biomass to fuel. As a result, each unit of biomass will produce more fuel. This means that dividing process costs by the energy content of the fuel results in dividing by a larger denominator. The only category where P-ZSM-5 underperforms the unmodified catalyst is in the generation of steam and power, where excess energy from coke combustion is used to produce steam. P-ZSM-5 produces less coke and therefore produces less excess heat during combustion.

**Figure 7:**
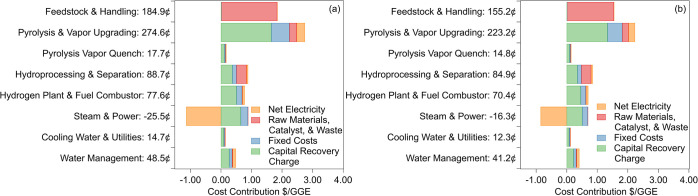
Contribution of each process step and utility to the final minimum fuel selling price for aviation fuel using (a) P-ZSM-5 and (b) ZSM-5 in the vapor upgrading step as determined by technoeconomic assessment.

Lifecycle assessment reveals that both catalysts offer substantial reductions in carbon intensity relative to fossil fuels. Specifically, the unmodified ZSM-5 achieved an 84% reduction in greenhouse gas emissions from the petroleum jet fuel baseline while P-ZSM-5 achieved an 80% reduction.^60^ The smaller reduction using P-ZSM-5 results from the smaller amount of steam and electricity generated using this catalyst. The lifecycle assessment uses the generated electricity to replace existing grid fossil sources and therefore assigns a larger greenhouse gas reduction to the unmodified material which produced more coke and therefore electricity. Figure S12 collects the contribution of each process step to the final GHG emissions. Beyond GHG emissions, the conceptual model was also used to quantify the rate of water consumption in the conversion process and determined that the P-ZSM-5 catalyst led to lower water use (2.44 gallons/GGE) than the unmodified ZSM-5 (2.79 gallons/GGE). This difference is driven primarily by the higher fuel production per unit of feedstock of the P-ZSM-5 catalyst, resulting in more efficient water usage. Future work is needed to better understand the implications of catalyst change on other sustainability metrics such as particulate matter or ozone depletion.

## Conclusions

In this work we demonstrate how phosphorus modification of ZSM-5 can reduce coking during CFP and increase the carbon efficiency of an integrated biomass to fuel pathway. A series of P-ZSM-5 catalysts were prepared via incipient wetness impregnation with phosphoric acid to achieve nominal P loadings of 1.0, 2.5, and 5.0 wt %. Catalyst characterization using NH_3_ TPD, pyridine FT-IR, UV Raman, and STEM-EDS confirmed the ability to effectively modulate acid site density to achieve values ranging from 0.77 mmol/g on unmodified ZSM-5 to 0.29 mmol/g on 5%-P-ZSM-5. Each catalyst was initially evaluated through a series of screening experiments using a microscale pyrolysis GCMS unit. The results revealed an increase in the production of desirable C_6_+ fuel range compounds over 2.5%-P-ZSM-5. These observations were supported by additional testing using an MBMS reactor system, which showed an increase in selectivity to deoxygenated hydrocarbons, particularly at high biomass to catalyst ratios. Based on these results, the 2.5%-P-ZSM-5 catalyst was down-selected for continuous reaction testing using a large bench-scale system comprised of a 2″ bubbling fluidized bed pyrolysis unit coupled to a downstream fluidized bed catalytic upgrading reactor. During these experiments the 2.5%-P-ZSM-5 catalyst exhibited a statistically significant reduction in coke (9.7 ± 0.2 vs 12.6 ± 0.4 C %) and a corresponding increase in bio-oil yield (25.6 ± 0.7 vs 22.3 ± 0.7 C %) compared to the unmodified ZSM-5 catalyst. The bio-oil generated during these experiments was subsequently hydrotreated and fractionated to evaluate the performance benefits within the context of an end-to-end pathway. These experiments revealed an 11% relative increase in the overall carbon yield from biomass to aviation fuel, which translates to a 14% decrease in the minimum fuel selling price as determined by technoeconomic analysis. Collectively, these data demonstrate the potential for a reduction in coke and increase in bio-oil yields through phosphorus modification of CFP catalysts and highlight the benefits of improved carbon efficiency to the overall process economics. Additional research is needed to provide a full assessment of catalyst durability over extended reaction periods and evaluate P doping in parallel with other chemical and structural modification strategies (e.g., SiO_2_:Al_2_O_3_ ratio) to achieve optimal zeolite properties.

## Supplementary Material



## Notes

Experimental details including feedstock composition, catalyst cost estimates, lifecycle analysis, catalyst characterization, and reaction testing results (PDF)
