# Supplementary material for: Reducing Coke and Increasing Bio-Oil Yield during Catalytic Fast Pyrolysis of Biomass Using Phosphorus-Modified Zeolite Catalysts
Source: ACS Sustain Chem Eng. 2025 Dec 3;13(49):21015–29. doi: 10.1021/acssuschemeng.5c04731 (PMC12709612; doi:10.1021/acssuschemeng.5c04731)
Supplement: Supplementary file 1 [file sc5c04731_si_001.pdf]

Supporting Information

Reducing coke and increasing bio-oil yield during catalytic fast pyrolysis of biomass using phosphorus modified zeolite catalysts

Cody J. Wrasman[1], Brittney E. Petel[1], Carson Pierce[1], Kellene A. Orton[1], Scott Palmer[1], Jacklyn N. Hall[2], Hacksung Kim[2,3], Fulya Dogan[2], Theodore R. Krause[2], Huamin Wang[4], Xinbin Yu[4], Stefano Dell'Orco[1], Kinga A. Unocic[5], Alexandre C. Foucher[5], Tomas Grejtak[5], David A. Cullen[5], Grace Blaskowski[1], Frederick G. Baddour[1], Xiaolin Chen[1], Kristiina Iisa[1], Abhijit Dutta[1], Susan E. Habas[1*], Michael B. Griffin[1*]

[1]National Renewable Energy Laboratory, Golden, CO 80401, USA
[2]Argonne National Laboratory, Lemont, IL 60439, USA
[3]Northwestern University, Evanston, IL 60208, USA
[4]Pacific Northwest National Laboratory, Richland, WA 99352, USA
[5]Oak Ridge National Laboratory, Oak Ridge, TN 37831, USA
*Michael.Griffin@nrel.gov
*Susan.Habas@nrel.gov

Contents

Table	Description	Page
S1	Ultimate and proximate analysis of the pine feedstock	2
S2	Masses tracked for product characterization in MBMS experiments	5
S3-S20	Catalyst cost estimate information	6-21
S21	CFP reaction testing carbon yield information	24
S22	CFP reaction testing mass yield information	24
S23	CFP reaction testing gas yield information	25
S24	Area fractions for Raman peak fitting	25
S25	Mass fractions detected by SEM-EDS in post-reaction catalysts	26
S26	Boiling point ranges for hydrotreated fuel fraction distillation	26
S27	Fuel property testing information	26

Figure	Description	Page
S1	Pine particle size distribution	2
S2	^{27}Al MAS NMR of the unmodified ZSM-5 catalyst	3
S3	N_2 physisorption plots for zeolite catalysts	3
S4	X-ray diffraction patterns for zeolite catalysts	4
S5	Hydrocarbon and pyrolysis vapor ion fraction from MBMS experiments	5
S6	NH_3 TPD plots of large batch zeolite catalysts	22
S7	Pyridine FT-IR spectra of large batch zeolite catalysts	22
S8	X-ray diffraction patterns of large batch zeolite catalysts	23
S9	N_2 physisorption of large batch zeolite catalysts	23
S10	^{31}P NMR spectra of post reaction zeolite catalysts	24
S11	Cross-sectional SEM-EDS of post-reaction zeolite catalysts	25
S12	Process emission information	27

Table S1 – Ultimate and proximate analysis of the pine feedstock.

Dry basis elemental composition [wt. %]	
C	51.3
H	6.1
O	40.9
N	0.1
Water content [wt. %]	5.9
Volatile content [wt. %]	77.3
Fixed carbon [wt. %]	15.3
Ash [wt. %]	1.5

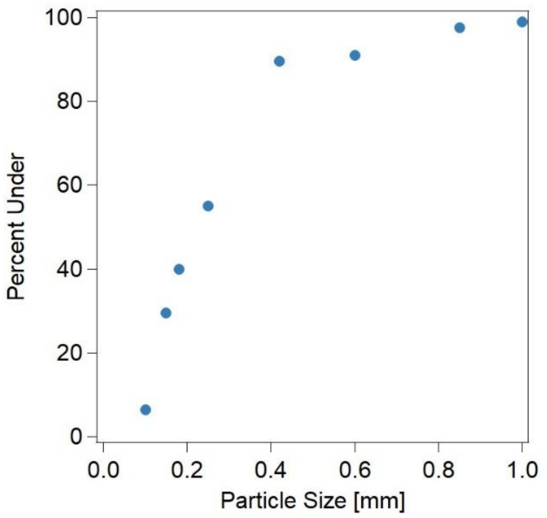

Figure S1 – Pine particle size distribution

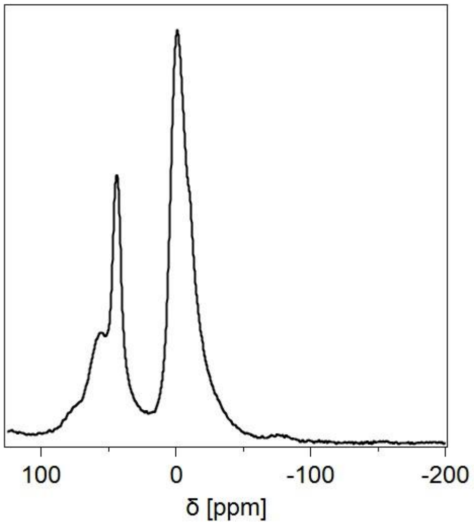

Figure S2 – ^{27}Al MAS NMR of the unmodified formed ZSM-5 catalyst.

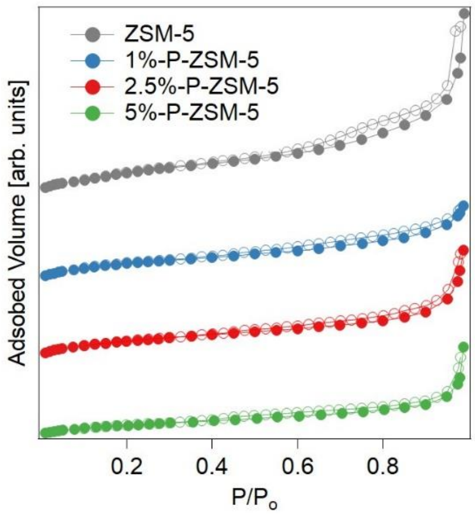

Figure S3 – N_2 physisorption plots for 1%-, 2.5%-, and 5%-P-ZSM-5 compared to unmodified ZSM-5. Filled symbols represent adsorption and empty symbols represent desorption.

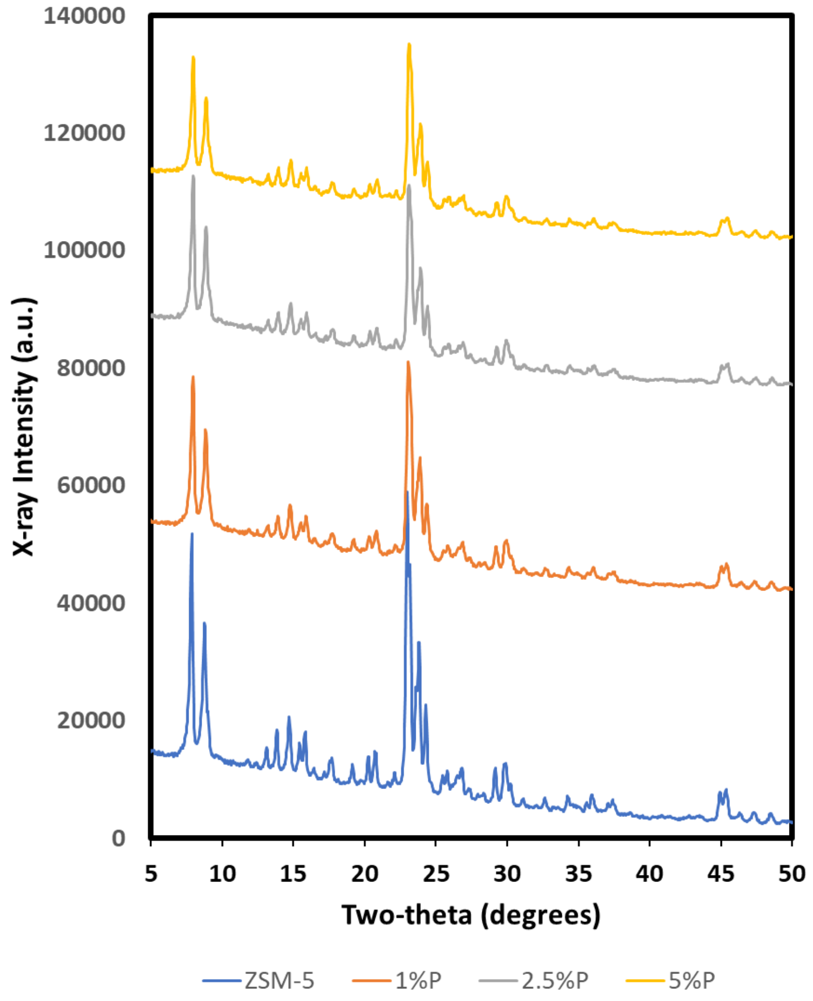

Figure S4 – X-ray diffraction (XRD) patterns for ZSM-5, 1%-P-ZSM-5, 2.5%-P-ZSM-5, 5%-P-ZSM-5. Sample XRD patterns are offset for clarity.

Table S2 – Lists of masses tracked for product characterization in MBMS

M/Z	Assignment
Hydrocarbons	
41	Propylene
42	Propylene
78	Benzene
91	Toluene
106	Xylenes
116	Indene
128	Naphthalene
Raw Pyrolysis Vapors	
29	Aldehydes
39	Furans
43	Aldehydes
55	Lignin fragments from Ref[1]
110	
137	
138	
150	
152	
164	

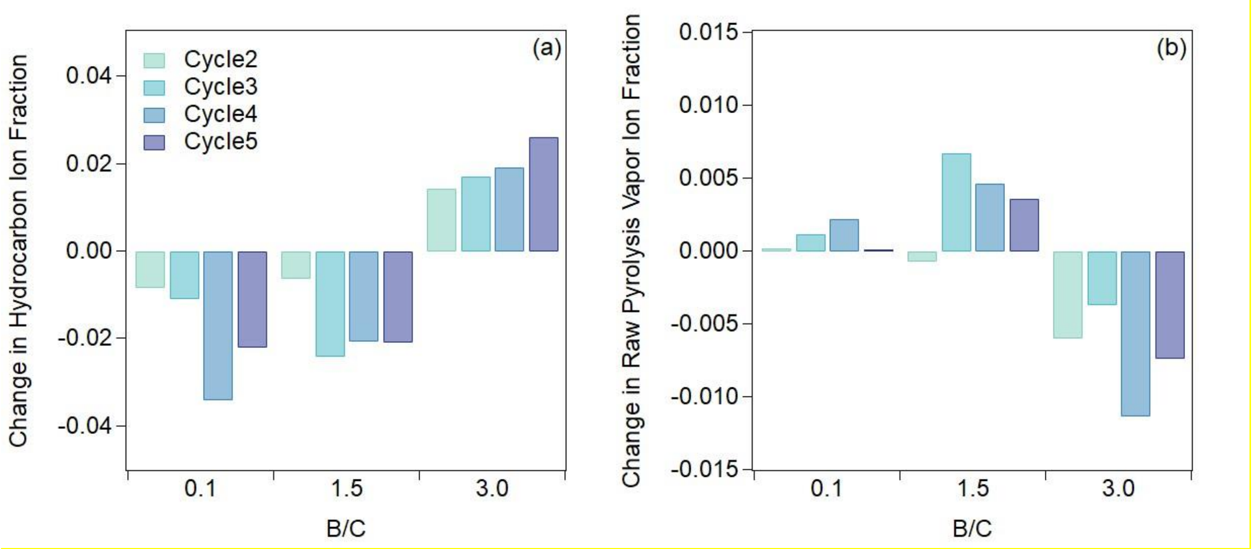

Figure S5 – Changes in (a) hydrocarbon and (b) raw pyrolysis vapor ion fraction for each regeneration cycle relative to the first cycle in Figure 3c-3d at B/C of 0.1, 1.5, and 3.0 for 2.5%-P-ZSM-5.

Table S3 – CatCost Inputs for the unmodified ZSM-5 catalyst.

CatCost v1.1.0 Summary – 1 Inputs

Estimate: ZSM-5 80% with Al2O3

Global Inputs		
Economics		
Basis Year	2016	
Currency	USD ($)	
Output Units		
Mass Unit	kg	
Processing Cost Estimation Inputs – Step Method		
Synthesis Campaign Size	Value	Units
Order size (1-1000 tons)	450	ton
Equipment size	Large (150 ton/day)	
Synthesis time	3	days
Cleaning time	1	days
Total: Synthesis campaign length	4	days
Overhead and Selling Margin		
G&A Overhead	5	%
SARD Overhead	5	%
Selling Margin	10.60	%
Processing Cost Estimation Inputs – CapEx & OpEx Factors		
Production Capacity - Catalyst	Value	Units
Design Production, Annual	8.26E+07	kg
Capacity Factor	100	%
Actual Production, Annual	8.26E+07	kg
Production Capacity - Operating Hours		
Operating Hours (Labor)	8760	hours
On-Stream Factor	90	%
Operating Hours (Production)	7884	hours
Design Production Rate	1.05E+04	kg/hr
Plant Life		
Plant Life (for depreciation)	10	years
Selling Margin		
Calculate using (select):	ROI	
Return on capital invested (pre-tax)	15	%/year
Flat margin (% of costs)		%

Table S4 – CatCost materials inputs for the unmodified ZSM-5 catalyst.

CatCost v1.1.0 Summary – 2 Materials

Estimate: ZSM-5 80% with Al2O3

Materials Calculation Inputs

Stoichiometric Calculations	Value	Units
Yield Type: % Yield or Mass?	AP Mass	
Finished Catalyst Mass Yield	1	kg
Stoichiometric Ratio AP/metal	1	mol/mol
Active Phase Molecular Weight	195.084	g/mol
Active Phase Weight Percent	80	%
Active Phase Mass at Prep Scale	1	kg
Catalyst Mass at Prep Scale	1.25	kg
Losses Due to Waste/Spoilage	3	%

Materials Cost Totals

Cost ($/kg catalyst)	Cost ($/year)
**2.2520**	**186,117,580**

*Outputs consider losses to waste/spoilage

Metal Sources

User Inputs			*Pricing Calculations*				*Outputs**	
Material Name	Quantity (Q)	Unit	Q in kg	Q in mol	Q (kg/kg catalyst)	Unit Price (2016 $/kg)	Cost ($/kg catalyst)	Cost ($/year)

Supports

User Inputs			*Pricing Calculations*				*Outputs**	
Material Name	Quantity (Q)	Unit	Q in kg	Q in mol	Q (kg/kg catalyst)	Unit Price (2016 $/kg)	Cost ($/kg catalyst)	Cost ($/year)
Alumina, metalurgical grade, bulk, spot	0.25	kg	0.2500	n/a	0.2000	2.32	0.4777	39,478,995

Other Materials

User Inputs			*Pricing Calculations*				*Outputs**	
Material Name	Quantity (Q)	Unit	Q in kg	Q in mol	Q (kg/kg catalyst)	Unit Price (2016 $/kg)	Cost ($/kg catalyst)	Cost ($/year)
Sodium silicate 41 BE	3.28	kg	3.2831	n/a	2.6265	0.55	1.4910	123,226,913
Al(OH)3	0.03	kg	0.0286	n/a	0.0229	0.66	0.0156	1,289,604
NaOH 50%	0.14	kg	0.1400	n/a	0.1120	0.44	0.0509	4,203,718
TPABr	0.01	kg	0.0080	n/a	0.0064	16.53	0.1090	9,007,968
HNO3 68%	0.17	kg	0.1660	n/a	0.1328	0.50	0.0679	5,607,460
NH4OH 28%	0.22	kg	0.2200	n/a	0.1760	0.22	0.0400	3,302,922

Table S5 – CatCost equipment requirements for the unmodified ZSM-5 catalyst.

CatCost v1.1.0 Summary – 3b Equipment, part 1: inputs

Estimate: ZSM-5 80% with Al2O3

Process Template or User-Entered Custom Process

Selected Process Template: Zeolite ZSM-5 (25%)

Scaling Inputs

Scaling Input Specific to this Process Design/Template

THIS PROCESS Design Production Rate 1.00E+08 lb catalyst/year

THIS PROCESS Design Production Rate, in Estimate Units 5.75E+03 catalyst/hour

From CatCost "1 Inputs" Sheet (Specific to this Estimate)

ESTIMATE Design Production Rate 1.05E+04 catalyst/hour

Equipment List: Inputs at This Process Design Production Rate

Equipment Type	User Label	Material of Construction	Quantity	Size	Size Unit
Reactor, jacketed, agitated	1	Carbon steel	2	11.36	volume, m3
Reactor, jacketed, agitated	2	Carbon steel	2	75.71	volume, m3
Crystallizer, batch evaporative	3	Carbon steel	6	4010.7	Volume, ft3
Filter, rotary-drum vacuum	4	Carbon steel and Polypropylene wetted parts	2	100	Filtering area, ft2
Reactor, jacketed, agitated	5	Carbon steel	1	5.68	volume, m3
Reactor, jacketed, agitated	6	Carbon steel	1	0.76	volume, m3
Evaporator, forced circulation	7	Carbon steel	1	600	Heat transfer area, ft2
Reactor, jacketed, agitated	8	Carbon steel	1	11.36	volume, m3
Dryer, indirect-heat steam-tube rotary (Seider)	9	Stainless steel	1	738.2742736	Heat-transfer area, ft2
Reactor, jacketed, agitated	10	Carbon steel	1	24.04	volume, m3
Reactor, jacketed, agitated	11	Carbon steel	1	28.39	volume, m3
Filter, rotary-drum vacuum	12	Carbon steel and Polypropylene wetted parts	1	100	Filtering area, ft2
Muller mixer	13	Carbon steel	3	100	Volume, ft3
Dryer, spray (Sinnott)	14	304 stainless steel	1	8645.4	evap rate kg/h
Reactor, jacketed, agitated	15	Carbon steel	1	1.14	volume, m3
Reactor, jacketed, agitated	16	Carbon steel	1	30.28	volume, m3
Filter, rotary-drum vacuum	17	Carbon steel and Polypropylene wetted parts	1	255	Filtering area, ft2
Reactor, jacketed, agitated	18	Rubber-lined	1	3.79	volume, m3
Reactor, jacketed, agitated	19	Rubber-lined	1	30.28	volume, m3
Filter, rotary-drum vacuum	20	Carbon steel and Polypropylene wetted parts	1	255	Filtering area, ft2
Dryer, steam-tube 2.4 m diameter, 350-1200 m2 (Perry's)	21	Carbon steel	1	2323.4	Free tube surface area, m2
Cooling tower w/ pumps, field assembled	OSBL	Carbon steel	2	1577	flow, L/s
ZSM-5 auxiliary equipment A: pumps, blowers, sm tanks	aux A	Standard	1	10	arbitrary units
ZSM-5 auxiliary equipment B: storage tanks	aux B	Standard	1	10	arbitrary units

Table S6 – CatCost equipment costs for the unmodified ZSM-5 catalyst.

CatCost v1.1.0 Summary – 3b Equipment, part 2: outputs

Estimate: ZSM-5 80% with Al2O3

Equipment Cost Totals		
**Purchase Cost**	**$**	**59,959,111**
**Installed Cost**	**$**	**99,897,145**
**Labor Factor (# of operators)**	**20.8**	

Equipment List: Scaling to Estimate Design Production Rate			*Equipment List: Outputs at Estimate Design Production Rate*		
Equipment Type	Quantity	Size	Purchase Cost	Installed Cost	Labor Factor
Reactor, jacketed, agitated	2	20.7	670,062	1,139,106	0.6
Reactor, jacketed, agitated	3	92.0	2,982,595	5,070,412	0.9
Crystallizer, batch evaporative	44	996.5	16,531,061	29,755,909	7.0
Filter, rotary-drum vacuum	2	182.2	527,566	891,587	0.4
Reactor, jacketed, agitated	1	10.3	212,894	361,920	0.3
Reactor, jacketed, agitated	1	1.4	81,061	137,803	0.3
Evaporator, forced circulation	1	1,093.2	393,526	822,469	0.3
Reactor, jacketed, agitated	1	20.7	335,031	569,553	0.3
Dryer, indirect-heat steam-tube rotary (Seider)	1	1,345.1	462,415	758,361	0.5
Reactor, jacketed, agitated	1	43.8	570,773	970,314	0.3
Reactor, jacketed, agitated	1	51.7	645,157	1,096,768	0.3
Filter, rotary-drum vacuum	1	182.2	263,783	445,794	0.2
Muller mixer	3	182.2	839,301	1,175,022	0.6
Dryer, spray (Sinnott)	4	3,938.0	5,988,678	9,821,431	4.0
Reactor, jacketed, agitated	1	2.1	93,694	159,280	0.3
Reactor, jacketed, agitated	1	55.2	676,750	1,150,475	0.3
Filter, rotary-drum vacuum	1	464.6	397,508	671,789	0.2
Reactor, jacketed, agitated	1	6.9	217,521	369,785	0.3
Reactor, jacketed, agitated	1	55.2	879,775	1,495,617	0.3
Filter, rotary-drum vacuum	1	464.6	397,508	671,789	0.2
Dryer, steam-tube 2.4 m diameter, 350-1200 m2 (Perry's)	4	1,058.3	6,838,694	11,215,458	2.0
Cooling tower w/ pumps, field assembled	2	2,873.3	4,297,246	5,156,695	1.2
ZSM-5 auxiliary equipment A: pumps, blowers, sm tanks	1	18.2	8,946,579	14,851,320	-
ZSM-5 auxiliary equipment B: storage tanks	1	18.2	6,709,932	11,138,486	-

Table S7 – CatCost utilities costs for the unmodified ZSM-5 catalyst.

CatCost v1.1.0 Summary – 3c Utilities

Estimate: ZSM-5 80% with Al2O3

Selected Process Template						
Selected Process Template:	Zeolite ZSM-5 (25%)					
Process Utilities Consumption and Cost for Selected Template						
Utility	Consumption	Units	Unit Cost	Units	Cost ($/kg catalyst)	Cost ($/year)
Cooling Water	0.341142686	kgal	0.14	$/kgal	0.0478	3,947,101
Process Water	0	kgal	1.3	$/kgal	-	-
Steam, 150 psig	0.00462253	ton	5	$/ton	0.0231	1,910,134
Steam, 600 psig	0	ton	5.7	$/ton	-	-
Electricity	0.499002073	kWh	0.055	$/kWh	0.0274	2,268,189
Natural Gas	0.017334486	MMBtu	3	$/MMBtu	0.0520	4,297,801

Utilities Cost Totals	
**Cost ($/kg catalyst)**	**Cost ($/year)**
**0.1503**	**12,423,225**

Table S8 – CatCost CapEx factors for unmodified ZSM-5 catalyst

CatCost v1.1.0 Summary – 3d CapEx

Estimate: ZSM-5 80% with Al2O3

CapEx & OpEx Factors: Factored Capital Expenditures

Cost Item	Value	Units	Total Cost ($)
Direct Capital			
Purchased Equipment	100	% of purchased equipment cost	59,959,111
Installation	67	% of purchased equipment cost	39,938,034
Instrumentation and Controls	26	% of purchased equipment cost	15,589,369
Piping	31	% of purchased equipment cost	18,587,324
Electrical	10	% of purchased equipment cost	5,995,911
Buildings	29	% of purchased equipment cost	17,388,142
Yard Improvements	12	% of purchased equipment cost	7,195,093
Service Facilities	55	% of purchased equipment cost	32,977,511
Waste Treatment	5	% of purchased equipment cost	2,997,956
Land	6	% of purchased equipment cost	3,597,547
**Total Direct**	**341**	**% of purchased equipment cost**	**204,225,998**
Indirect Capital			
Engineering and Supervision	32	% of purchased equipment cost	19,186,915
Construction Expenses	34	% of purchased equipment cost	20,386,098
Legal Expenses	4	% of purchased equipment cost	2,398,364
Contractor's Fee	19	% of purchased equipment cost	11,392,231
Contingency	37	% of purchased equipment cost	22,184,871
**Total Indirect**	**126**	**% of purchased equipment cost**	**75,548,480**
**Total Fixed Capital Investment (FCI)**	**467**	**% of purchased equipment cost**	**279,774,478**
Working Capital	75	% of purchased equipment cost	44,969,333
**Total Capital Investment (TCI)**	**542**	**% of purchased equipment cost**	**324,743,811**

Table S9 – CatCost operating expenses inputs for the unmodified ZSM-5 catalyst.

CatCost v1.1.0 Summary – 3e OpEx

Estimate: ZSM-5 80% with Al2O3

CapEx & OpEx Factors: Factored Operating Expenditures

Cost Item	Value	Units	Cost ($/year)	Cost ($/kg catalyst)
Direct Labor				
Direct Labor Operators (rounded up)	21	operators		
Direct Labor Hours per Year	183,960	hr/yr		
Direct Labor Rate	48	$/hr		
**Direct Labor Cost (DL)**			**8,830,080**	**0.1068**
Direct Operating Costs				
Supervisory and Clerical Labor	18	% of DL	1,589,414	0.0192
Laboratory Charges	15	% of DL	1,324,512	0.0160
Maintenance and Repair (M&R)	5	% of FCI	13,988,724	0.1693
Operating Supplies	15	% of M&R	2,098,309	0.0254
**Total: Labor, Supplies, Maintenance, Lab (LSM)**			**27,831,039**	**0.3368**
Fixed/Indirect Operating Costs				
Local Taxes	2.5	% of FCI	6,994,362	0.0846
Insurance	0.8	% of FCI	2,238,196	0.0271
Rent, % of value of rented land	10	% of land	359,755	0.0044
Plant Overhead, % of LSM	60	% of LSM	16,698,623	0.2021
**Total: Taxes, Insurance, Rent, Overhead**			**26,290,936**	**0.3181**
General Expenses				
Administration	20	% of LSM	5,566,208	0.0674
Distribution and Marketing	10	% of op. costs excluding PGM/noble metals content	25,266,278	0.3057
Research and Development	5	% of op. costs excluding PGM/noble metals content	12,633,139	0.1529
**Total: Admin, Dist., Mkting., R&D**			**43,465,625**	**0.5259**

Table S10 – CatCost spent catalyst value calculation for the unmodified ZSM-5 catalyst.

CatCost v1.1.0 Summary – 4 Spent Catalyst

Estimate: ZSM-5 80% with Al2O3

Spent Catalyst Value		
Inputs	Value	Units
Metal to recover	Aluminum	
Support	SiO2	
Metal wt. % of AP	3	%
Catalyst bulk density	780	kg/m3
Planned reactor configuration	Slurry/Fluidized Bed	
Has trace Sn, Cu, Fe > 2% of AP?	No	
Classification for Sale or Landfill	Zeolite, Silica, Alumina	
Catalyst Attrition During Use	Value	Units
Active Phase Losses		
AP mass in fresh catalyst	0.8000	kg/kg catalyst
Metal mass in fresh catalyst	0.0240	kg/kg catalyst
AP losses during use (typical)	4	%
AP mass after use	0.7680	kg/kg catalyst
Metal mass after use	0.0230	kg/kg catalyst
Support Losses		
Support mass in fresh catalyst	0.2000	kg/kg catalyst
Support losses during use (typical)	2	%
Support mass after use	0.1960	kg/kg catalyst
Total Catalyst Solids		
Catalyst mass after use	0.9640	kg/kg catalyst
Catalyst volume after use	0.0436	ft3/kg catalyst
Metals Recovery Value and Fees		Units
Value of Metal Content		
Metal losses during refining (typical)	70	%
Recoverable metal	0.0069	kg/kg catalyst
Recoverable metal, troy ounces	0.2222	oz t/kg catalyst
Spot price escalated to basis year	1.95	$/kg metal
**Recoverable metal value**	**0.01**	**$/kg catalyst**
Costs of Recovery		
Incoming fee	5.04	$/kg catalyst
Thermal oxidation fee	0.29	$/kg catalyst
Refining fee	-	$/kg catalyst
Metal contaminant fee (Sn, Cu, Fe)	-	$/kg catalyst
**Total recovery fees**	5.33	$/kg catalyst
Landfill Fees and Sale Value		
**Landfill cost**	**(0.37)**	**$/kg catalyst**
**Sale value, if any**	**0.11**	**$/kg catalyst**
**Best Choice for this Spent Catalyst:**	**Sale**	
**Total Spent Catalyst Value/(Cost)**	**0.11**	**$/kg catalyst**

Table S11 – CatCost final cost output data for the unmodified ZSM-5 catalyst.

CatCost v1.1.0 – 5 Outputs, CapEx & OpEx Factors

Estimate: ZSM-5 80% with Al2O3

General Output Parameters		
Unit Cost in Cents or Dollars (USD, $)	Dollars	
Annual, Monthly, Weekly, Daily Cost?	Annual	

Estimate Details		
Basis Year	2016	
Design Production, Annual	8.26E+07 kg	
Actual Production, Annual	8.26E+07 kg	

CapEx & OpEx Factors Outputs		
**Cost Item**	**Unit Cost**	**Annual Cost**
	**$/kg catalyst**	**$/year**
**Capital Costs (10-year plant life)**		
Fixed Capital Investment	0.3385	27,977,448
Working Capital	0.0544	4,496,933
**Total Capital Investment**	**0.3929**	**32,474,381**
**Operating Costs**		
Direct Operating Costs		
Raw Materials	2.2520	186,117,580
Process Utilities	0.1503	12,423,225
Labor, Supplies, Maintenance, Lab	0.3368	27,831,039
Indirect Operating Costs		
Taxes, Insurance, Rent, Overhead	0.3181	26,290,936
General Expenses		
Admin, Dist., Mkting., R&D	0.5259	43,465,625
**Total Operating Costs**	**3.5832**	**296,128,404**
**Selling Margin**		
Return on Capital Investment		
(15%/yr of total capital invested)	0.5894	48,711,572
Flat Margin (disabled)	-	-
**Total Margin**	**0.5894**	**48,711,572**
**Catalyst Purchase Cost**	**4.5655**	**377,314,356**
**Spent Catalyst Value (SCV)**	**0.1063**	**8,782,040**
**Net Catalyst Cost**	**4.4592**	**368,532,316**

Table S12 – CatCost Inputs for the 2.5%-P-ZSM-5 catalyst.

CatCost v1.1.0 Summary – 1 Inputs

Estimate: P-ZSM-5 80% with Al2O3

Global Inputs		
Economics		
Basis Year	2016	
Currency	USD ($)	
Output Units		
Mass Unit	kg	
Processing Cost Estimation Inputs – Step Method		
Synthesis Campaign Size	Value	Units
Order size (1-1000 tons)	450	ton
Equipment size	Large (150 ton/day)	
Synthesis time	3	days
Cleaning time	1	days
Total: Synthesis campaign length	4	days
Overhead and Selling Margin		
G&A Overhead	5	%
SARD Overhead	5	%
Selling Margin	10.60	%
Processing Cost Estimation Inputs – CapEx & OpEx Factors		
Production Capacity - Catalyst	Value	Units
Design Production, Annual	8.26E+07	kg
Capacity Factor	100	%
Actual Production, Annual	8.26E+07	kg
Production Capacity - Operating Hours		
Operating Hours (Labor)	8760	hours
On-Stream Factor	90	%
Operating Hours (Production)	7884	hours
Design Production Rate	1.05E+04	kg/hr
Plant Life		
Plant Life (for depreciation)	10	years
Selling Margin		
Calculate using (select):	ROI	
Return on capital invested (pre-tax)	15	%/year
Flat margin (% of costs)		%

Table S13 – CatCost materials inputs for the 2.5%-P-ZSM-5 catalyst.

CatCost v1.1.0 Summary – 2 Materials

Estimate: P-ZSM-5 80% with Al2O3

Materials Calculation Inputs		
Stoichiometric Calculations	Value	Units
Yield Type: % Yield or Mass?	AP Mass	
Finished Catalyst Mass Yield	1	kg
Stoichiometric Ratio AP/metal	1	mol/mol
Active Phase Molecular Weight	195.084	g/mol
Active Phase Weight Percent	80	%
Active Phase Mass at Prep Scale	1	kg
Catalyst Mass at Prep Scale	1.25	kg
Losses Due to Waste/Spoilage	3	%

Materials Cost Totals	
**Cost ($/kg catalyst)**	**Cost ($/year)**
**2.3450**	**193,799,428**

*Outputs consider losses to waste/spoilage

Metal Sources

User Inputs			*Pricing Calculations*				*Outputs**	
Material Name	Quantity (Q)	Unit	Q in kg	Q in mol	Q (kg/kg catalyst)	Unit Price (2016 $/kg)	Cost ($/kg catalyst)	Cost ($/year)

Supports

User Inputs			*Pricing Calculations*				*Outputs**	
Material Name	Quantity (Q)	Unit	Q in kg	Q in mol	Q (kg/kg catalyst)	Unit Price (2016 $/kg)	Cost ($/kg catalyst)	Cost ($/year)
Alumina, metalurgical grade, bulk, spot	0.25	kg	0.2500	n/a	0.2000	2.32	0.4777	39,478,995

Other Materials

User Inputs			*Pricing Calculations*				*Outputs**	
Material Name	Quantity (Q)	Unit	Q in kg	Q in mol	Q (kg/kg catalyst)	Unit Price (2016 $/kg)	Cost ($/kg catalyst)	Cost ($/year)
Sodium silicate 41 BE	3.28	kg	3.2831	n/a	2.6265	0.55	1.4910	123,226,913
Al(OH)3	0.03	kg	0.0286	n/a	0.0229	0.66	0.0156	1,289,604
NaOH 50%	0.14	kg	0.1400	n/a	0.1120	0.44	0.0509	4,203,718
TPABr	0.01	kg	0.0080	n/a	0.0064	16.53	0.1090	9,007,968
HNO3 68%	0.17	kg	0.1660	n/a	0.1328	0.50	0.0679	5,607,460
NH4OH 28%	0.22	kg	0.2200	n/a	0.1760	0.22	0.0400	3,302,922
Phosphoric acid, 85% tanks, frt. equald.	0.10	kg	0.1000	n/a	0.0800	1.13	0.0930	7,681,848

Table S14 – CatCost equipment requirements for the 2.5%-P-ZSM-5 catalyst.

CatCost v1.1.0 Summary – 3b Equipment, part 1: inputs

Estimate: P-ZSM-5 80% with Al2O3

Process Template or User-Entered Custom Process

Selected Process Template:	Zeolite ZSM-5 (25%)

Scaling Inputs

Scaling Input Specific to this Process Design/Template		
THIS PROCESS Design Production Rate	1.00E+08	lb catalyst/year
THIS PROCESS Design Production Rate, in Estimate Units	5.75E+03	catalyst/hour
From CatCost "1 Inputs" Sheet (Specific to this Estimate)		
ESTIMATE Design Production Rate	1.05E+04	catalyst/hour

Equipment List: Inputs at This Process Design Production Rate

Equipment Type	User Label	Material of Construction	Quantity	Size	Size Unit
Reactor, jacketed, agitated	1	Carbon steel	2	11.36	volume, m3
Reactor, jacketed, agitated	2	Carbon steel	2	75.71	volume, m3
Crystallizer, batch evaporative	3	Carbon steel	6	4010.7	Volume, ft3
Filter, rotary-drum vacuum	4	Carbon steel and Polypropylene wetted parts	2	100	Filtering area, ft2
Reactor, jacketed, agitated	5	Carbon steel	1	5.68	volume, m3
Reactor, jacketed, agitated	6	Carbon steel	1	0.76	volume, m3
Evaporator, forced circulation	7	Carbon steel	1	600	Heat transfer area, ft2
Reactor, jacketed, agitated	8	Carbon steel	1	11.36	volume, m3
Dryer, indirect-heat steam-tube rotary (Seider)	9	Stainless steel	1	738.2742736	Heat-transfer area, ft2
Reactor, jacketed, agitated	10	Carbon steel	1	24.04	volume, m3
Reactor, jacketed, agitated	11	Carbon steel	1	28.39	volume, m3
Filter, rotary-drum vacuum	12	Carbon steel and Polypropylene wetted parts	1	100	Filtering area, ft2
Muller mixer	13	Carbon steel	3	100	Volume, ft3
Dryer, spray (Sinnott)	14	304 stainless steel	1	8645.4	evap rate kg/h
Reactor, jacketed, agitated	15	Carbon steel	1	1.14	volume, m3
Reactor, jacketed, agitated	16	Carbon steel	1	30.28	volume, m3
Filter, rotary-drum vacuum	17	Carbon steel and Polypropylene wetted parts	1	255	Filtering area, ft2
Reactor, jacketed, agitated	18	Rubber-lined	1	3.79	volume, m3
Reactor, jacketed, agitated	19	Rubber-lined	1	30.28	volume, m3
Filter, rotary-drum vacuum	20	Carbon steel and Polypropylene wetted parts	1	255	Filtering area, ft2
Dryer, steam-tube 2.4 m diameter, 350-1200 m2 (Perry's)	21	Carbon steel	1	2323.4	Free tube surface area, m2
Cooling tower w/ pumps, field assembled	OSBL	Carbon steel	2	1577	flow, L/s
ZSM-5 auxiliary equipment A: pumps, blowers, sm tanks	aux A	Standard	1	10	arbitrary units
ZSM-5 auxiliary equipment B: storage tanks	aux B	Standard	1	10	arbitrary units

Table S15 - Table S6 – CatCost equipment costs for the 2.5%-P-ZSM-5 catalyst.

CatCost v1.1.0 Summary – 3b Equipment, part 2: outputs

Estimate: P-ZSM-5 80% with Al2O3

Equipment Cost Totals		
**Purchase Cost**	**$**	**59,959,111**
**Installed Cost**	**$**	**99,897,145**
**Labor Factor (# of operators)**	**20.8**	

Equipment List: Scaling to Estimate Design Production Rate			*Equipment List: Outputs at Estimate Design Production Rate*		
Equipment Type	Quantity	Size	Purchase Cost	Installed Cost	Labor Factor
Reactor, jacketed, agitated	2	20.7	670,062	1,139,106	0.6
Reactor, jacketed, agitated	3	92.0	2,982,595	5,070,412	0.9
Crystallizer, batch evaporative	44	996.5	16,531,061	29,755,909	7.0
Filter, rotary-drum vacuum	2	182.2	527,566	891,587	0.4
Reactor, jacketed, agitated	1	10.3	212,894	361,920	0.3
Reactor, jacketed, agitated	1	1.4	81,061	137,803	0.3
Evaporator, forced circulation	1	1,093.2	393,526	822,469	0.3
Reactor, jacketed, agitated	1	20.7	335,031	569,553	0.3
Dryer, indirect-heat steam-tube rotary (Seider)	1	1,345.1	462,415	758,361	0.5
Reactor, jacketed, agitated	1	43.8	570,773	970,314	0.3
Reactor, jacketed, agitated	1	51.7	645,157	1,096,768	0.3
Filter, rotary-drum vacuum	1	182.2	263,783	445,794	0.2
Muller mixer	3	182.2	839,301	1,175,022	0.6
Dryer, spray (Sinnott)	4	3,938.0	5,988,678	9,821,431	4.0
Reactor, jacketed, agitated	1	2.1	93,694	159,280	0.3
Reactor, jacketed, agitated	1	55.2	676,750	1,150,475	0.3
Filter, rotary-drum vacuum	1	464.6	397,508	671,789	0.2
Reactor, jacketed, agitated	1	6.9	217,521	369,785	0.3
Reactor, jacketed, agitated	1	55.2	879,775	1,495,617	0.3
Filter, rotary-drum vacuum	1	464.6	397,508	671,789	0.2
Dryer, steam-tube 2.4 m diameter, 350-1200 m2 (Perry's)	4	1,058.3	6,838,694	11,215,458	2.0
Cooling tower w/ pumps, field assembled	2	2,873.3	4,297,246	5,156,695	1.2
ZSM-5 auxiliary equipment A: pumps, blowers, sm tanks	1	18.2	8,946,579	14,851,320	-
ZSM-5 auxiliary equipment B: storage tanks	1	18.2	6,709,932	11,138,486	-

Table S16 – CatCost utilities costs for the 2.5%-P-ZSM-5 catalyst.

CatCost v1.1.0 Summary – 3c Utilities

Estimate: P-ZSM-5 80% with Al2O3

Selected Process Template	
Selected Process Template:	Zeolite ZSM-5 (25%)

Process Utilities Consumption and Cost for Selected Template

Utility	Consumption	Units	Unit Cost	Units	Cost ($/kg catalyst)	Cost ($/year)
Cooling Water	0.341142686	kgal	0.14	$/kgal	0.0478	3,947,101
Process Water	0	kgal	1.3	$/kgal	-	-
Steam, 150 psig	0.00462253	ton	5	$/ton	0.0231	1,910,134
Steam, 600 psig	0	ton	5.7	$/ton	-	-
Electricity	0.499002073	kWh	0.055	$/kWh	0.0274	2,268,189
Natural Gas	0.017334486	MMBtu	3	$/MMBtu	0.0520	4,297,801

Utilities Cost Totals	
**Cost ($/kg catalyst)**	**Cost ($/year)**
**0.1503**	**12,423,225**

Table S17 – CatCost CapEx factors for the 2.5%-P-ZSM-5 catalyst

CatCost v1.1.0 Summary – 3d CapEx

Estimate: P-ZSM-5 80% with Al2O3

CapEx & OpEx Factors: Factored Capital Expenditures

Cost Item	Value	Units	Total Cost ($)
Direct Capital			
Purchased Equipment	100	% of purchased equipment cost	59,959,111
Installation	67	% of purchased equipment cost	39,938,034
Instrumentation and Controls	26	% of purchased equipment cost	15,589,369
Piping	31	% of purchased equipment cost	18,587,324
Electrical	10	% of purchased equipment cost	5,995,911
Buildings	29	% of purchased equipment cost	17,388,142
Yard Improvements	12	% of purchased equipment cost	7,195,093
Service Facilities	55	% of purchased equipment cost	32,977,511
Waste Treatment	5	% of purchased equipment cost	2,997,956
Land	6	% of purchased equipment cost	3,597,547
**Total Direct**	**341**	**% of purchased equipment cost**	**204,225,998**
Indirect Capital			
Engineering and Supervision	32	% of purchased equipment cost	19,186,915
Construction Expenses	34	% of purchased equipment cost	20,386,098
Legal Expenses	4	% of purchased equipment cost	2,398,364
Contractor's Fee	19	% of purchased equipment cost	11,392,231
Contingency	37	% of purchased equipment cost	22,184,871
**Total Indirect**	**126**	**% of purchased equipment cost**	**75,548,480**
**Total Fixed Capital Investment (FCI)**	**467**	**% of purchased equipment cost**	**279,774,478**
Working Capital	75	% of purchased equipment cost	44,969,333
**Total Capital Investment (TCI)**	**542**	**% of purchased equipment cost**	**324,743,811**

Table S18 - CatCost operating expenses inputs for the 2.5%-P-ZSM-5 catalyst.

CatCost v1.1.0 Summary – 3e OpEx

Estimate: P-ZSM-5 80% with Al2O3

CapEx & OpEx Factors: Factored Operating Expenditures				
Cost Item	Value	Units	Cost ($/year)	Cost ($/kg catalyst)
Direct Labor				
Direct Labor Operators (rounded up)	21	operators		
Direct Labor Hours per Year	183,960	hr/yr		
Direct Labor Rate	48	$/hr		
**Direct Labor Cost (DL)**			**8,830,080**	**0.1068**
Direct Operating Costs				
Supervisory and Clerical Labor	18	% of DL	1,589,414	0.0192
Laboratory Charges	15	% of DL	1,324,512	0.0160
Maintenance and Repair (M&R)	5	% of FCI	13,988,724	0.1693
Operating Supplies	15	% of M&R	2,098,309	0.0254
**Total: Labor, Supplies, Maintenance, Lab (LSM)**			**27,831,039**	**0.3368**
Fixed/Indirect Operating Costs				
Local Taxes	2.5	% of FCI	6,994,362	0.0846
Insurance	0.8	% of FCI	2,238,196	0.0271
Rent, % of value of rented land	10	% of land	359,755	0.0044
Plant Overhead, % of LSM	60	% of LSM	16,698,623	0.2021
**Total: Taxes, Insurance, Rent, Overhead**			**26,290,936**	**0.3181**
General Expenses				
Administration	20	% of LSM	5,566,208	0.0674
Distribution and Marketing	10	% of op. costs excluding PGM/noble metals content	26,034,463	0.3150
Research and Development	5	% of op. costs excluding PGM/noble metals content	13,017,231	0.1575
**Total: Admin, Dist., Mkting., R&D**			**44,617,902**	**0.5399**

Table S19 - CatCost spent catalyst value calculation for the 2.5%-P-ZSM-5 catalyst.

CatCost v1.1.0 Summary – 4 Spent Catalyst

Estimate: P-ZSM-5 80% with Al2O3

Spent Catalyst Value		
Inputs	Value	Units
Metal to recover	Aluminum	
Support	SiO2	
Metal wt. % of AP	3	%
Catalyst bulk density	780	kg/m3
Planned reactor configuration	Slurry/Fluidized Bed	
Has trace Sn, Cu, Fe > 2% of AP?	No	
Classification for Sale or Landfill	Zeolite, Silica, Alumina	
Catalyst Attrition During Use	Value	Units
Active Phase Losses		
AP mass in fresh catalyst	0.8000	kg/kg catalyst
Metal mass in fresh catalyst	0.0240	kg/kg catalyst
AP losses during use (typical)	4	%
AP mass after use	0.7680	kg/kg catalyst
Metal mass after use	0.0230	kg/kg catalyst
Support Losses		
Support mass in fresh catalyst	0.2000	kg/kg catalyst
Support losses during use (typical)	2	%
Support mass after use	0.1960	kg/kg catalyst
Total Catalyst Solids		
Catalyst mass after use	0.9640	kg/kg catalyst
Catalyst volume after use	0.0436	ft3/kg catalyst
Metals Recovery Value and Fees		Units
Value of Metal Content		
Metal losses during refining (typical)	70	%
Recoverable metal	0.0069	kg/kg catalyst
Recoverable metal, troy ounces	0.2222	oz t/kg catalyst
Spot price escalated to basis year	1.95	$/kg metal
**Recoverable metal value**	**0.01**	**$/kg catalyst**
Costs of Recovery		
Incoming fee	5.04	$/kg catalyst
Thermal oxidation fee	0.29	$/kg catalyst
Refining fee	-	$/kg catalyst
Metal contaminant fee (Sn, Cu, Fe)	-	$/kg catalyst
**Total recovery fees**	5.33	$/kg catalyst
Landfill Fees and Sale Value		
**Landfill cost**	**(0.37)**	**$/kg catalyst**
**Sale value, if any**	**0.11**	**$/kg catalyst**
**Best Choice for this Spent Catalyst:**	**Sale**	
**Total Spent Catalyst Value/(Cost)**	**0.11**	**$/kg catalyst**

Table S20 - CatCost final cost output data for the 2.5%-P-ZSM-5 catalyst.

CatCost v1.1.0 – 5 Outputs, CapEx & OpEx Factors

Estimate: P-ZSM-5 80% with Al2O3

General Output Parameters		
Unit Cost in Cents or Dollars (USD, $)	Dollars	
Annual, Monthly, Weekly, Daily Cost?	Annual	
Estimate Details		
Basis Year	2016	
Design Production, Annual	8.26E+07 kg	
Actual Production, Annual	8.26E+07 kg	
CapEx & OpEx Factors Outputs		
Cost Item	Unit Cost	Annual Cost
	**$/kg catalyst**	**$/year**
**Capital Costs (10-year plant life)**		
Fixed Capital Investment	0.3385	27,977,448
Working Capital	0.0544	4,496,933
**Total Capital Investment**	**0.3929**	**32,474,381**
**Operating Costs**		
Direct Operating Costs		
Raw Materials	2.3450	193,799,428
Process Utilities	0.1503	12,423,225
Labor, Supplies, Maintenance, Lab	0.3368	27,831,039
Indirect Operating Costs		
Taxes, Insurance, Rent, Overhead	0.3181	26,290,936
General Expenses		
Admin, Dist., Mkting., R&D	0.5399	44,617,902
**Total Operating Costs**	**3.6901**	**304,962,529**
**Selling Margin**		
Return on Capital Investment		
(15%/yr of total capital invested)	0.5894	48,711,572
Flat Margin (disabled)	-	-
**Total Margin**	**0.5894**	**48,711,572**
**Catalyst Purchase Cost**	**4.6724**	**386,148,482**
**Spent Catalyst Value (SCV)**	**0.1063**	**8,782,040**
**Net Catalyst Cost**	**4.5661**	**377,366,442**

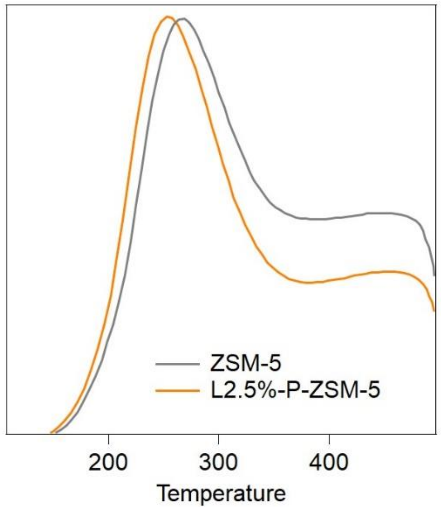

Figure S6 – NH_3 TPD plots collected for unmodified ZSM-5 as well as L2.5%-P-ZSM-5.

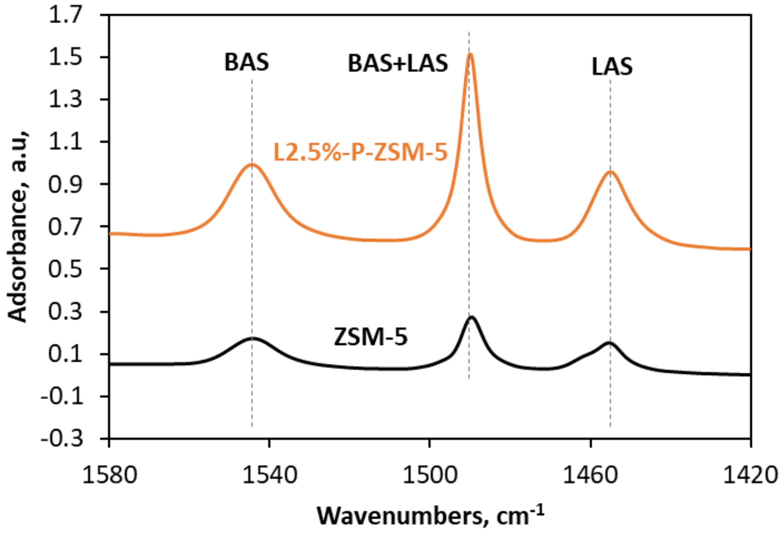

Figure S7 - IR spectra of pyridine adsorption on the ZSM-5 and L2.5%-P-ZSM-5 (BAS and LAS denote pyridine adsorbed on Bronsted acid and Lewis acid sites, respectively).

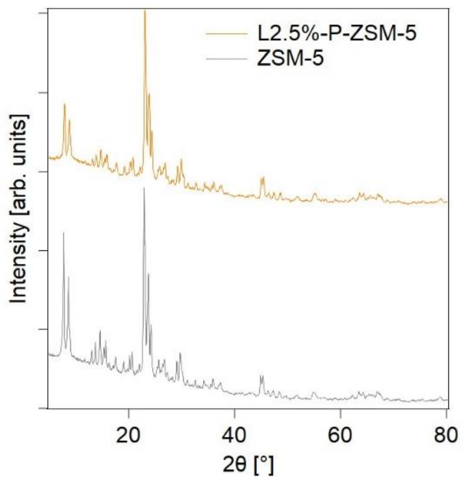

Figure S8 – X-ray diffraction of unmodified ZSM-5 and L2.5%-P-ZSM-5. Sample XRD patterns are offset for clarity.

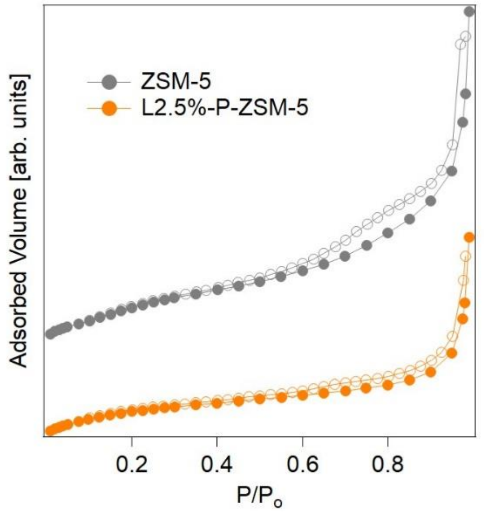

Figure S9 – N_2 physisorption plots for unmodified ZSM-5 and L2.5%-P-ZSM-5. Filled symbols represent adsorption and empty symbols represent desorption.

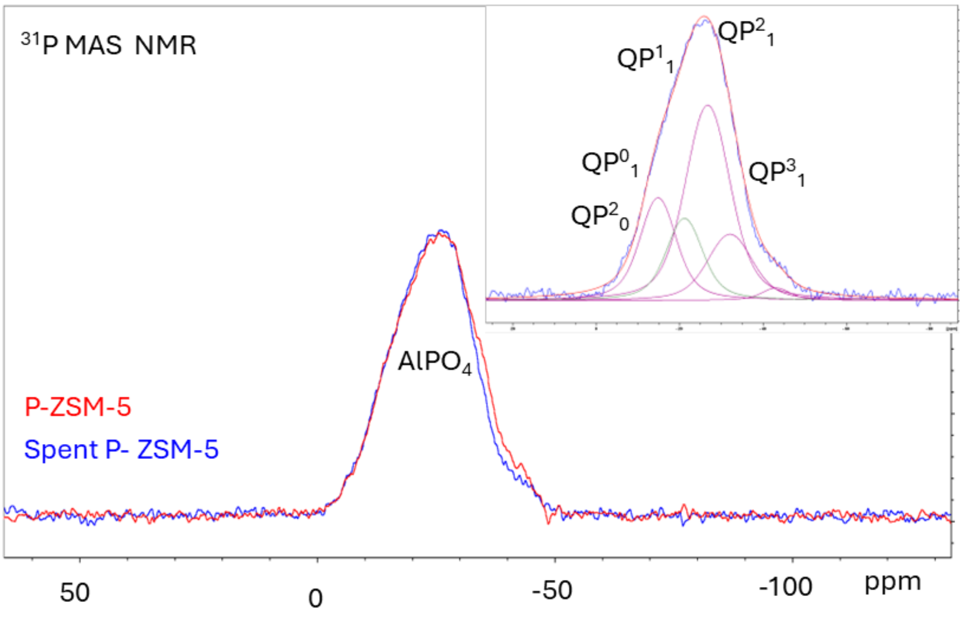

Figure S10 – ^{31}P NMR spectrum for P-ZSM-5 before and after CFP in the 2FBR system.

Table S21 – Carbon yields displayed in Figure 5. Uncertainties represent the standard deviation of three repeated experiments.

Fraction of dry feedstock C [% C]	ZSM-5	L2.5%-P-ZSM-5
Bio-oil	22.3 ± 0.7	25.6 ± 0.7
Aqueous	6.1 ± 0.3	5.7 ± 0.4
Condensable Gases	7 ± 1	7.2 ± 0.4
Permanent Gases	23.8 ± 0.2	22.7 ± 0.3
Char	20 ± 2	20.7 ± 0.7
Coke	12.6 ± 0.4	9.7 ± 0.2
Balance	92 ± 2	92 ± 1

Table S22 – Mass yields on a dry biomass basis for the data displayed in Figure 5. Uncertainties represent the standard deviation of three repeated experiments.

Mass yield [g/$g_{biomass}$]	ZSM-5	L2.5%-P-ZSM-5
Organic	17.1 ± 0.4	19.7 ± 0.5
Aqueous	25.8 ± 0.4	23.3 ± 0.6
Condensable gases	5.2 ± 0.7	5.3 ± 0.3
Light gases	27.4 ± 0.2	26.4 ± 0.4
Water Vapor	0.7 ± 0.1	0.7 ± 0.1
Char	13 ± 1	14.1 ± 0.2
Coke	6.1 ± 0.4	6.1 ± 0.9
Balance	95 ± 1	95.6 ± 0.6

Table S23 – Permanent gas carbon yields. Uncertainties represent the standard deviation of triplicate experiments.

Carbon Yields [%]	ZSM-5	L2.5%-P-ZSM-5
CO	13.63 ± 0.08	13.6 ± 0.2
CO_2	7.86 ± 0.09	7.5 ± 0.1
Alkanes	1.69 ± 0.01	1.59 ± 0.05
Alkenes	2.61 ± 0.02	2.25 ± 0.05

Table S24 – Area fractions represented by each fitted peak in Figure 6c-d Raman spectra as well as the fraction of all observed area represented by the Raman G band.

	ZSM-5	L2.5%-P-ZSM-5
Peak 1 Area Fraction	0.15	0.11
Peak 2 Area Fraction	0.17	0.28
Peak 3 Area Fraction	0.02	0.08
Peak 4 Area Fraction	0.44	0.06
Peak 5 Area Fraction	0.21	0.47
G Band Fraction (Peaks 4+ Peak 5)	0.65	0.53

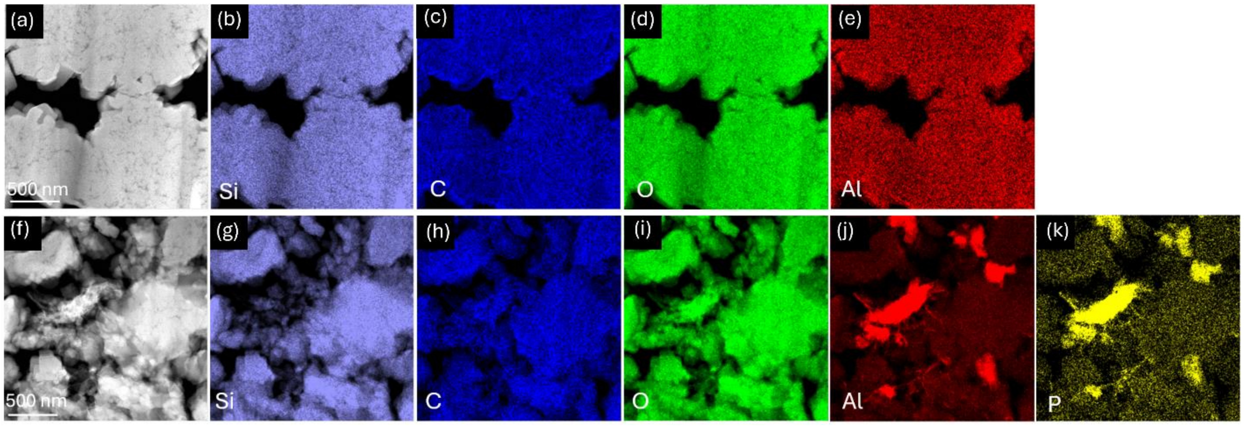

Figure S11 – Cross-sectional STEM-EDS of (a-e) spent 5-cycle ZSM-5 and (f-k) spent 5-cycle P-ZSM-5.

Table S25 – Mass fraction of elements detected by SEM-EDS in spent 5-cycle ZSM-5 and spent 5-cycle P-ZSM-5, including and avoiding regions containing alumina binder.

	Mass Fraction (%)			
	ZSM-5		P-ZSM-5	
Element	With binder	Avoiding binder	With binder	Avoiding binder
C	13.11	14.01	10.48	6.95
O	24.97	54.44	59.16	56.01
Al	5.20	1.10	7.47	1.34
Si	56.72	30.45	20.50	35.50
P	0	0	2.39	0.20

Table S26 – Boiling point ranges for hydrotreated fuel fraction distillation.

Fraction	Atmospheric equivalent boiling point range [°C]
Gasoline	<110
Jet	110 – 275
Diesel	275 – 330
Residue	>330

Table S27 – ASTM 4054 guidelines for jet fuel as well as the measured values for hydrotreated jet fuel produced from oil made using ZSM-5 and L2.5%-P-ZSM-5.

Property	ASTM 4054	ZSM-5	L2.5%-P-ZSM-5
Density at 15 °C [g/mL]	0.730 – 0.880	0.844	0.852
Flash point [°C]	>38	34.7	33.7
Freeze point [°C]	<-40	<-75	<-75
Viscosity at -20 °C [mm^2/s]	<8	3.9	3.4
Surface tension at 22 °C [mN/m]	25 – 29	27.6	27.8
Lower Heating Value [MJ/kg]	>42.8	41.7	42.4

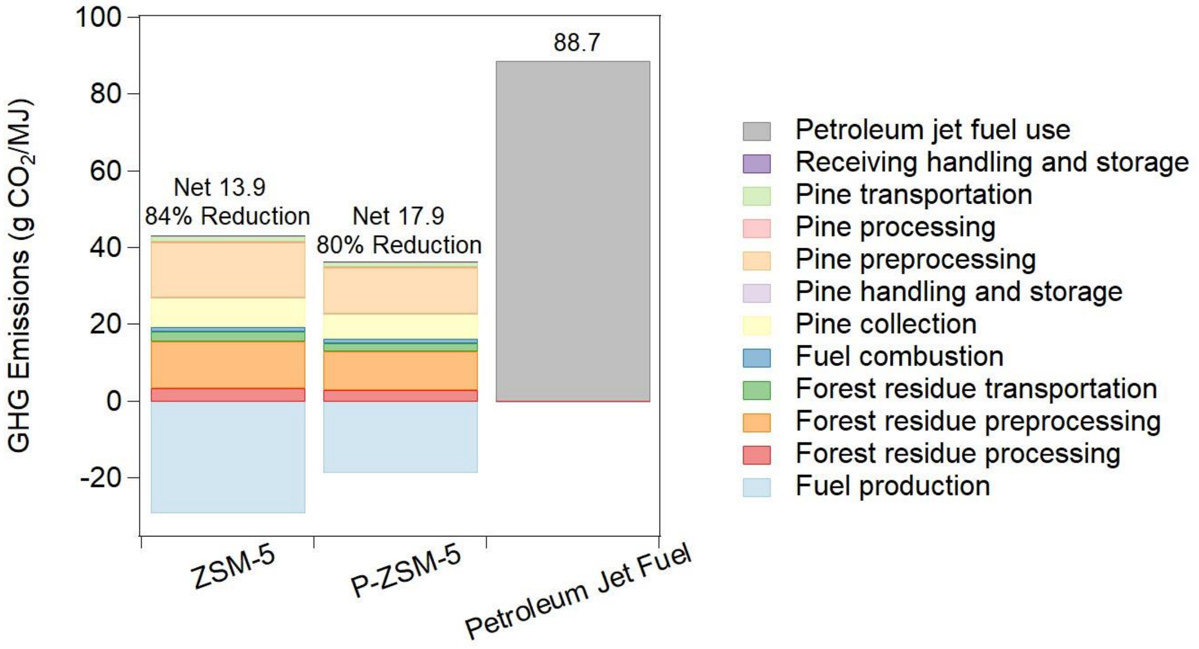

Figure S12 – GHG emissions contributions and credits for the ZSM-5 and 2.5%-P-ZSM-5 modeled cases.

References

(1) Mukarakate, C.; Zhang, X.; R. Stanton, A.; J. Robichaud, D.; N. Ciesielski, P.; Malhotra, K.; S. Donohoe, B.; Gjersing, E.; J. Evans, R.; S. Heroux, D.; Richards, R.; Iisa, K.; R. Nimlos, M. Real-Time Monitoring of the Deactivation of HZSM-5 during Upgrading of Pine Pyrolysis Vapors. *Green Chemistry* **2014**, *16* (3), 1444–1461. https://doi.org/10.1039/C3GC42065E.
